# Polygenic Risk Profiles and the Manifestations of Alcohol Use Disorder and Related Outcomes

**DOI:** 10.1111/acer.70390

**Published:** 2026-10-12

**Authors:** Genevieve F. Dash, Fazil Aliev, Sally I‐Chun Kuo, Andrey Anokhin, Sarah J. Brislin, Kathleen Bucholz, Grace Chan, Maia Choi, Emma C. Johnson, Chella Kamarajan, Samuel Kuperman, Alex P. Miller, John I. Nurnberger, Martin H. Plawecki, Holly E. Poore, John Rice, Leah Wetherill, COGA Collaborators, B. Porjesz, V. Hesselbrock, A. Agrawal, H. J. Edenberg, T. Foroud, Y. Liu, M. H. Plawecki, S. Kuperman, A. Anderson, B. Porjesz, J. Meyers, L. Bierut, A. Agrawal, S. Hartz, M. Schuckit, D. Dick, R. Hart, J. Salvatore, J. Tischfield, A. Goate, P. Slesinger, C. Holzhauer, M. Hesselbrock, D. Lai, J. Nurnberger, L. Wetherill, X. Xuei, S. O'Connor, J. Kramer, G. Chan, C. Kamarajan, A. Pandey, D. B. Chorlian, P. Barr, S. Kinreich, G. Pandey, Z. Neale, C. Chatzinakos, J. Zhang, Saenz deViteri, R. Christian, A. Bingly, G. Pathak, A. Anokhin, K. Bucholz, F. Dong, A. Hatoum, E. Johnson, V. McCutcheon, J. Rice, S. Saccone, F. Aliev, Z. Pang, S. Kuo, S. Brislin, J. Moore, A. Merikangas, M. Gitik, Danielle M. Dick

**Affiliations:** ^1^ Department of Psychiatry Rutgers Robert Wood Johnson Medical School Piscataway New Jersey USA; ^2^ Rutgers Addiction Research Center, Brain Health Institute Piscataway New Jersey USA; ^3^ Department of Psychiatry WashU School of Medicine St. Louis Missouri USA; ^4^ Department of Psychiatry UConn Health Farmington Connecticut USA; ^5^ Department of Psychiatry University of Iowa Carver College of Medicine Iowa City Iowa USA; ^6^ Department of Psychology Rutgers University New Brunswick New Jersey USA; ^7^ Department of Psychiatry SUNY Downstate Health Sciences University Brooklyn New York USA; ^8^ Department of Psychiatry Indiana University School of Medicine Indianapolis Indiana USA; ^9^ Department of Medical and Molecular Genetics Indiana University School of Medicine Indianapolis Indiana USA

**Keywords:** alcohol use disorder, COGA, depression, externalizing, polygenic scores

## Abstract

**Background:**

Research suggests that multiple genetically influenced pathways of risk impact alcohol use disorder (AUD), including externalizing (disinhibition, behavioral undercontrol), internalizing (negative affect, depression), and alcohol‐specific (physiological response to alcohol) factors. This study examined the joint effects of genetic influences representing these risk domains on AUD and their influence on comorbid phenotypes among individuals with AUD.

**Methods:**

Participants of European‐like ancestry from the Collaborative Study on the Genetics of Alcoholism (*N* = 6883) provided data on lifetime AUD, conduct disorder (CD), antisocial personality disorder (ASPD), major depressive disorder (MDD), alcohol expectancies, sensation seeking, and neuroticism. Polygenic scores (PGS) representing externalizing (EXT), major depressive disorder (MDD), and alcohol‐specific (ALC‐sp) risk were calculated. First, participants were grouped based on combinations of high versus low (top vs. bottom 30%) EXT, MDD, and ALC‐sp PGS (*n* = 2006), and AUD outcomes were compared across groups. Second, among individuals with AUD, psychopathology, alcohol expectancies, sensation seeking, and neuroticism were compared across those with high versus low EXT (*n* = 1664), MDD (*n* = 1620), and ALC‐sp (*n* = 1669) PGS.

**Results:**

Participants with three high PGS and those with high EXT + high ALC‐sp PGS had the most severe AUD outcomes. High EXT + high MDD PGS and high MDD + high ALC‐sp PGS groups generally did not differ from groups with only one high PGS. Comparisons between groups with only one high PGS were not significant. Among individuals with AUD, high EXT PGS was associated with CD/ASPD, arousal/aggression alcohol expectancies, and relaxation/tension reduction alcohol expectancies; high MDD PGS was associated with MDD and neuroticism.

**Conclusions:**

Quantity and type of genetic risk are important for AUD outcomes. EXT plays an outsized role in AUD, particularly in the presence of other high PGS, and may potentiate alcohol expectancies that reinforce harmful patterns of use. Findings elucidate the role of multiple pathways of genetic risk for AUD and how they impact comorbid phenotypes.

## Graphical abstract

There are multiple genetically‐influenced pathways of risk that influence the development of alcohol use disorder (AUD), including externalizing (EXT; disinhibition, behavioral undercontrol), internalizing (INT; negative affect, depression), and alcohol‐specific (ALC‐sp; physiological response to alcohol) factors. Findings from this study show that both the quantity and types of genetic risk factors a person carries are important in influencing AUD outcomes, with EXT genetic risk playing an outsized role in AUD, particularly in the presence of high INT and ALC‐sp genetic risk.

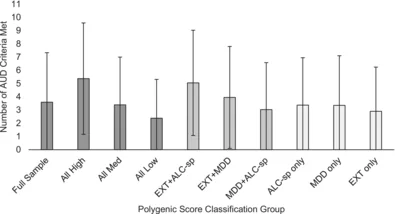

## 1 Introduction

Alcohol use remains a leading contributor to preventable and premature mortality in the United States (García et al. 2024) and worldwide (World Health Organization 2024). Alcohol use disorder (AUD) is approximately 50% heritable, meaning that roughly half of the variation across individuals in the likelihood of developing clinically significant alcohol‐related problems is attributable to genetic influences (Deak and Johnson 2021). In addition to demonstrating that genetic influences are important, twin and family studies have also provided information about *how* genetic influences impact AUD outcomes. Genetic influences on alcohol problems appear to operate largely through a general liability toward externalizing behavior (Poore et al. 2023, 2026), which influences many behaviors and disorders related to self‐regulation, including childhood conduct problems, general risk‐taking, and substance use disorders (Karlsson Linnér et al. 2021). To a lesser extent, alcohol problems also appear to share genetic influences with internalizing outcomes, such as depression and negative affect (Tully and Iacono 2016). These findings map onto the developmental literature, which also documents externalizing and internalizing pathways that have roots early in childhood and are associated with elevated risk for alcohol problems (Dick et al. 2013; Hussong et al. 2011, 2017; Zucker 2008). There is also evidence that genetic factors directly impact level of response to alcohol, which is also heritable (Schuckit 2018; Wall et al. 2016; Yeung et al. 2024) and associated with the development of alcohol‐related problems (Schuckit et al. 2009). In addition, there is known variation in genes involved in alcohol metabolism that impact the likelihood of developing AUD (Wall et al. 2016).

Large‐scale genome‐wide associations studies (GWAS) have now made it possible to map genetic variants involved in these pathways of externalizing, internalizing, and alcohol‐specific polygenic risk. A recent multivariate genomic analysis of ~2.2 million individuals (Poore et al. 2026) identified 708 significant genetic loci associated with alcohol problems via a general liability toward externalizing (EXT), and another 14 loci with alcohol‐specific (ALC‐sp) effects. With respect to internalizing risk, a study of over 1.1 million people identified 223 independent single nucleotide polymorphisms (SNPs) associated with major depressive disorder (MDD) (Levey et al. 2021). From these well‐powered GWAS, polygenic scores (PGS) that represent genome‐wide genetic liability can be calculated. PGS are created by summing risk alleles corresponding to a particular phenotype in each individual, each weighted by the effect size estimate and significance from the discovery GWAS (Choi et al. 2020). PGS can, in turn, be applied to independent samples to quantify risk for a phenotype of interest (e.g., externalizing, internalizing, alcohol response) at the individual level.

### 1.1 Present Study

Although EXT, MDD, and ALC‐sp PGS each independently account for variance in alcohol‐related outcomes (Poore et al. 2026; Zhou et al. 2017), individuals can carry risk across multiple genetic risk pathways. To our knowledge, no studies have yet examined the joint effect of these three genetic liabilities on alcohol outcomes and related phenotypes that often precede and/or co‐occur alongside AUD. Differences in the degree to which individuals carry genetic liability across multiple pathways that influence AUD and its comorbidities may contribute to widely observed heterogeneity in presentations of AUD; for example, drinking for externalizing reasons (e.g., enhancement) versus internalizing reasons (e.g., coping) (Cooper 1994; LaBrie et al. 2014; Savage et al. 2023; Tarter et al. 1994; Zucker 2008). Thus, the first aim of this study was to evaluate the influence of varying combinations of EXT, MDD, and ALC‐sp PGS on AUD outcomes by classifying participants based on their relative levels of EXT, MDD, and ALC‐sp PGS (e.g., high‐high‐high, high‐low‐high, low‐high‐high) and subsequently comparing AUD criterion count and diagnosis prevalence across groups. The second aim was to examine whether individuals who meet criteria for moderate or severe Diagnostic and Statistical Manual of Mental Disorders (DSM‐5) AUD (i.e., 4+ criteria met) and carry different combinations of genetic risk differ in their comorbidities and phenotypic presentation, including psychopathology (conduct disorder, antisocial personality disorder, major depressive disorder), alcohol expectancies, and personality traits (sensation seeking, neuroticism). Mirroring the approach taken in the first aim, participants were classified based on their relative levels of EXT, MDD, and ALC‐sp PGS and phenotypes of interest were compared across classification groups. Specific hypotheses are delineated alongside the corresponding analyses in the Analytic Plan. This study was preregistered on the Open Science Framework (OSF) (https://doi.org/10.17605/OSF.IO/X89U2).

## 2 Materials and Methods

### 2.1 Participants and Procedure

A sample of *N* = 6883 individuals age 18 years or older (52.56% female; M_age_ = 37.41 [SD = 14.51]) with available PGS data and of European‐like ancestry from the Collaborative Study on the Genetics of Alcoholism (COGA) was used for analyses (see Table 1 for sample characteristics). COGA employs a multigenerational, family‐based survey design. Individuals with AUD (probands) and their family members were recruited from treatment centers, along with a set of comparison families from the same communities. There have been four principal data collection waves: (1) initial ascertainment and assessment (1991–1999), (2) follow‐up of individuals in wave 1 and other family members not assessed at wave 1, as well as families from a new research site (1996–2005), (3) a “prospective” wave assessing offspring ages 12–22 from wave 1 and 2 case and comparison families in 2‐year intervals (2004–2019), and (4) a “lifespan” wave reassessing previous participants who are now in mid‐ and later‐life (2019‐present). Data were collated across waves. For each variable, we used data from the wave in which the greatest number of criteria was met (i.e., for participants with more than 1 assessment point, the maximum score for a given variable across timepoints was taken). Recruitment, data collection, and data management procedures and protocols for COGA are detailed extensively elsewhere (https://cogastudy.org) (Agrawal et al. 2023; Dick et al. 2023). Original data collection was approved by Institutional Review Boards at all data collection sites and secondary analysis of these deidentified data was considered exempt.

**TABLE 1:** Characteristics of the full sample and the polygenic score (PGS) classification group subsample.

Variables	Full sample				PGS classification group subsample			
	Total (*N* = 6883)	Male (*n* = 3265)	Female (*n* = 3618)	Avail. *N*	Total (*N* = 2006)	Male (*n* = 970)	Female (*n* = 1036)	Avail. *N*
Psychopathology (lifetime diagnosis)	%	%	%	*N*	%	%	%	*N*
DSM‐5 alcohol use disorder	59.33	72.04	47.87	6883	58.23	69.69	47.49	2006
2–3 Criteria (mild)	19.65	19.81	19.52	6883	19.19	19.48	18.92	2006
4–5 Criteria (moderate)	12.15	14.06	10.42	6883	11.57	13.92	9.36	2006
6+ Criteria (severe)	27.52	38.16	17.93	6883	27.47	36.29	19.21	2006
DSM‐IV conduct disorder	10.60	16.62	5.31	5944	10.85	16.51	5.56	1742
DSM‐IV antisocial personality disorder	9.63	16.11	3.90	6720	9.80	16.06	4.04	1948
DSM‐IV major depressive disorder	21.66	14.89	27.51	5539	21.96	17.04	26.45	1612

Criterion count (lifetime maximum)	M (SD)	M (SD)	M (SD)	*N*	M (SD)	M (SD)	M (SD)	*N*
DSM‐5 alcohol use disorder	3.61 (3.68)	4.65 (3.80)	2.67 (3.29)	6883	3.59 (3.74)	4.53 (3.85)	2.72 (3.40)	2006
DSM‐IV conduct disorder	0.91 (1.30)	1.25 (1.53)	0.61 (0.97)	5944	0.93 (1.33)	1.28 (1.54)	0.61 (1.00)	1742
DSM‐IV antisocial personality disorder	2.00 (1.76)	2.66 (1.78)	1.40 (1.51)	6875	1.99 (1.76)	2.65 (1.79)	1.36 (1.49)	2002
DSM‐IV major depressive disorder	3.83 (3.69)	3.00 (3.55)	4.66 (3.65)	1222	3.77 (3.70)	2.85 (3.53)	4.77 (3.64)	361

Alcohol expectancies	M (SD)	M (SD)	M (SD)	*N*	M (SD)	M (SD)	M (SD)	*N*
Physical/Social pleasure	30.67 (7.46)	31.84 (7.06)	29.66 (7.64)	5157	30.57 (7.52)	31.74 (7.30)	29.24 (7.57)	1506
Arousal/Aggression	26.97 (7.36)	27.64 (7.08)	26.40 (7.54)	5154	26.85 (7.37)	27.64 (7.07)	26.13 (7.57)	1505
Social assertiveness	32.02 (10.18)	33.21 (9.52)	31.01 (10.61)	5155	32.07 (9.99)	33.23 (9.32)	31.01 (10.45)	1505
Relaxation/tension reduction	27.96 (8.01)	29.44 (7.50)	26.68 (8.21)	5150	27.85 (8.01)	29.34 (7.37)	26.49 (8.32)	1504

Personality	M (SD)	M (SD)	M (SD)	*N*	M (SD)	M (SD)	M (SD)	*N*
Sensation seeking	15.52 (4.93)	16.66 (4.38)	14.01 (5.21)	394	15.33 (4.71)	16.17 (4.60)	14.04 (4.65)	111
Neuroticism (normed score)	52.48 (11.29)	52.99 (11.08)	52.04 (11.45)	5166	52.59 (11.30)	53.25 (10.88)	51.99 (11.65)	1512

### 2.2 Measures

#### 2.2.1 Alcohol Use Disorder and Psychopathology

AUD and other psychopathology were assessed using the Semi‐Structured Assessment for the Genetics of Alcoholism (SSAGA) (Bucholz et al. 1994). The SSAGA assesses DSM‐based diagnostic criteria for a range of psychopathology, including lifetime substance use disorders, antisocial personality disorder (ASPD), and major depressive disorder (MDD). As conduct disorder in childhood is a necessary feature of ASPD, conduct disorder (CD) assessment is embedded within and can be derived from the ASPD module. Lifetime substance use disorder and other psychopathology were indexed in two ways: a count index reflecting the number of criteria met, and a binary index reflecting the presence or absence of disorder. Due to data availability, AUD phenotypes were based on DSM‐5 assessments and the remaining psychopathology phenotypes were based on DSM‐IV assessments.

#### 2.2.2 Alcohol Expectancies

Alcohol expectancies were assessed using the Alcohol Expectancy Questionnaire (AEQ) (Brown et al. 1987). The AEQ is a 90‐item measure that captures six dimensions of alcohol expectancies, including four of interest here: physical and social pleasure (e.g., “having a few drinks is a nice way to celebrate special occasions”; *α* = 0.87), arousal and aggression (e.g., “after a few drinks it is easier to pick a fight”; *α* = 0.85), increased social assertiveness (e.g., “a few drinks make it easier to talk to people”; *α* = 0.93), and relaxation and tension reduction (e.g., “alcohol makes me worry less”; *α* = 0.90). Items were rated on a 1–5 Likert scale ranging from disagree strongly (1) to agree strongly (5). Alcohol expectancies were indexed by sum scores calculated for each dimension of interest. Missing items were prorated prior to summing for analysis, such that scales missing no more than 20% of items were scored by multiplying the sum of answered items by the total scale length and subsequently dividing by the number of non‐missing items in that scale.

#### 2.2.3 Sensation Seeking

Sensation seeking was assessed using the Zuckerman Sensation Seeking Scale (SSS) (Zuckerman et al. 1964; Zuckerman 2007). The SSS is a 40‐item measure, with each item consisting of two contrasting statements from which the respondent must choose the one that best describes them. One item in each pair reflects trait sensation seeking, such that response to each pair results in a score of 0 or 1. The SSS captures four dimensions of sensation seeking: disinhibition (e.g., “I like ‘wild uninhibited parties” OR “I prefer quiet parties with good conversation”), boredom susceptibility (e.g., “I get very restless if I have to stay around home for any length of time” OR “I enjoy spending time in the familiar surroundings of home”), thrill and adventure seeking, (e.g., “I sometimes like to do things that are a little frightening” OR “A sensible person avoids activities that are dangerous”), and experience seeking (“I like to explore a strange city or section of town by myself, even if it means getting lost” OR “I prefer a guide when I am in a place I don't know well”). Sensation‐seeking was indexed by the total score across domains. Missing items were prorated prior to summing for analysis using the same approach described above. Internal consistency was acceptable (*α* = 0.74).

#### 2.2.4 Neuroticism

Neuroticism was assessed using the Five‐Factor Personality Inventory (NEO‐FFI) (Costa and McCrae 1992). The neuroticism subscale of the NEO‐FFI is a 12‐item measure that captures tendency toward negative emotionality and psychological distress (e.g., a, “I often feel helpless and want someone else to solve my problems,” “I often feel tense and jittery”). Items are rated on a 1–5 Likert scale ranging from strongly disagree (1) to strongly agree (5). Neuroticism scores were indexed as normed *t* values.

#### 2.2.5 Polygenic Scores

COGA genotyping, imputation, and quality control procedures are detailed extensively elsewhere (Johnson et al. 2023). A Bayesian regression and continuous shrinkage method was used to create genome‐wide PGS (PRS‐CS) (Ge et al. 2019). The PRS‐CS method uses linkage disequilibrium (LD) information from the 1000 Genomes Project reference panel with matching ancestry and estimates the posterior effect sizes for SNPs in a given set of GWAS summary statistics. Discovery GWAS used for PGS construction included summary statistics from the largest GWAS for externalizing (Karlsson Linnér et al. 2021; Poore et al. 2026), depression (Adams et al. 2025), and alcohol‐specific genetic effects (genetic variation in alcohol use disorder after accounting for genetic variation in other externalizing phenotypes) (Poore et al. 2026) that were available upon initiation of analyses; all of these discovery GWAS were conducted in individuals of European‐like ancestry. Depression was used to capture internalizing because, at the time of analysis, there was no broad‐spectrum internalizing PGS derived from a multivariate GWAS of internalizing phenotypes that mirrored the multivariate externalizing PGS. As the PRS‐CS method is currently restricted to the ~1.3 million SNPs in the high‐quality consensus genotype set defined by The International HapMap 3 Consortium (2010), PGS were generated only using HapMap 3 SNPs. All other parameters were set to the default parameters of the PRS‐CS software, in which the global shrinkage parameter is set to Phi = 1e‐2 and the Markov Chain Monte Carlo (MCMC) parameters include 1000 total iterations, with the first 500 discarded as burn‐in, and every 5th iteration retained. Final PGS based on posterior PRS‐CS weights were computed in individuals assigned to a European genetic similarity group as estimated by their genetic data using the LD‐adjusted beta weights and calculated using the *score* procedure in PLINK 1.9 (Chang et al. 2015). PGS were *z*‐scored for analysis.

### 2.3 Analytic Plan

Analyses were conducted in SAS 9.4. The first set of analyses predicted AUD outcomes (number of criteria met, diagnosis) from classification groups indexing varying combinations of high and low scores on the three PGS. The second set of analyses was conducted in a subsample of individuals with lifetime AUD and tested for differences in psychopathology, alcohol expectancies, and personality traits across groups indexing varying combinations of high and low scores on EXT and MDD PGS. Individuals were considered to have AUD if they met 4 or more criteria (i.e., moderate or severe AUD), as the 2‐criterion (mild) threshold is often argued to be too low and thus overly inclusive of individuals without significant clinical impairment (Martin et al. 2011). Sensitivity analyses using the lower threshold of 2 or more criteria (i.e., any AUD severity) are reported in the Supporting Information. Each of the analyses and associated hypotheses are described in detail below.

#### 2.3.1 Predicting Alcohol Use Disorder Outcomes From EXT, MDD, and ALC‐sp PGS

Groups were created by categorizing subjects based on their relative levels of polygenic risk for EXT, MDD, and ALC‐sp (e.g., high‐high‐high, high‐low‐high, etc.). Individuals were considered “high,” “medium,” or “low” on a PGS if their score was in the top 30%, middle 40%, and bottom 30%, respectively. The groups of interest were individuals with any combination of “high” and “low” scores across the three PGS. The group with “medium” scores on all three PGS was included as a mean‐level comparator. This grouping procedure resulted in 27 PGS classification groups total, nine of which were of interest due to having combinations of “high” and “low” levels of PGS or “medium” levels of all three PGS. PGS classification groups were dummy coded and entered as predictors of the criterion count and diagnosis outcomes. We chose to analyze the data in this way to allow for intuitive comparisons of outcomes among individuals with varying combinations of genetic risk types. To evaluate differences in AUD mean criterion count and prevalence of AUD diagnosis across groups, estimated marginal means (least squares means) and pairwise comparisons were generated using the *diff* command within the PROC GENMOD *lsmeans* statement. Within these analyses, it was hypothesized that: (1) individuals with three high PGS would have the highest mean AUD criterion count and rates of AUD compared to all other groups, (2) individuals with two high PGS would show elevated mean criterion counts and rates of AUD compared to those with 1 high or all low PGS, (3) individuals with one high PGS would show elevated mean AUD criterion counts and rates of AUD compared to those with low scores on all three PGS, and (4) individuals with high EXT or ALC‐sp PGS, but low MDD PGS, would show higher mean AUD criterion count sand rates of AUD compared to individuals with high MDD but low EXT and ALC‐sp PGS, based on the rationale that ALC and EXT risk dimensions are more consistently associated with AUD outcomes. To account for the large number of comparisons conducted within this particular set of analyses (36 per outcome), false discovery rate (FDR) adjustment was applied using the Benjamini–Hochberg procedure (Benjamini and Hochberg 1995).

#### 2.3.2 Predicting Psychopathology, Alcohol Expectancies, and Personality Traits in the AUD Subsample

Individuals meeting criteria for lifetime AUD were designated as having high (top 30%) or low (bottom 30%) EXT PGS, MDD PGS, and ALC‐sp PGS. Again, we chose to analyze the data in this way to allow for comparisons of outcomes among individuals with varying combinations of genetic risk types. Mirroring the approach for modeling AUD outcomes, PGS categories (high vs. low EXT PGS; high vs. low MDD PGS; high vs. low ALC‐sp PGS) were used to predict psychopathology, alcohol expectancies, and personality traits in separate GLMs with group comparisons generated within the model. It was hypothesized that (1) individuals with AUD who have high EXT PGS would have higher rates of comorbid CD/ASPD, higher endorsement of physical/social pleasure and arousal/aggression alcohol expectancies, and higher levels of sensation seeking as compared to those with low EXT PGS; (2) that individuals with AUD who have high MDD PGS would have higher rates of comorbid depression, higher endorsement of social assertiveness and relaxation/tension reduction alcohol expectancies, and higher neuroticism as compared to those with low MDD PGS; and (3) that individuals with AUD who have high ALC‐sp PGS would not differ from those with low ALC‐sp PGS with respect to psychopathology, and no directional hypotheses were posited for alcohol expectancies.

#### 2.3.3 General Analytic Considerations

For all analyses, age, sex, and first 10 ancestry principal components (PCs) were included as covariates. All GLMs were fit in SAS PROC GENMOD and employed robust sandwich standard errors to account for familial clustering within the COGA data. A negative binomial distribution with a log link function was employed to model the criterion count variables due to skewness and overdispersion; a binomial distribution with a logit link function was employed to model the binary diagnosis variables. Model coefficients were exponentiated to produce incidence rate ratios (IRRs) and odds ratios (ORs) for negative binomial and logistic models, respectively. All other outcomes approximated distributional normality and were modeled accordingly. In addition to the models described above, analyses that preserved the continuous nature of the PGS and examined their two‐ and three‐way interactions were run and are presented in the Supporting Information.

## 3 Results

### 3.1 Descriptive Statistics

Descriptive statistics in the full sample (*N* = 6883) and PGS group classification subsample (*N* = 2006) are presented in Table 1. The average number of AUD criteria met was 3.61 (SD = 3.68) in the full sample and 3.59 (SD = 3.74) in the PGS classification group subsample (see Figure 1a). For both the full sample and subsample, nearly 60% met criteria for AUD of any severity (2+ criterion threshold), nearly 40% met criteria for moderate or severe AUD (4+ criterion threshold), and approximately 28% met criteria for severe AUD (6+ criterion threshold; see Figure 1b). Correlations between study variables for both the full sample and subsample are presented in Table 2.

**FIGURE 1:**
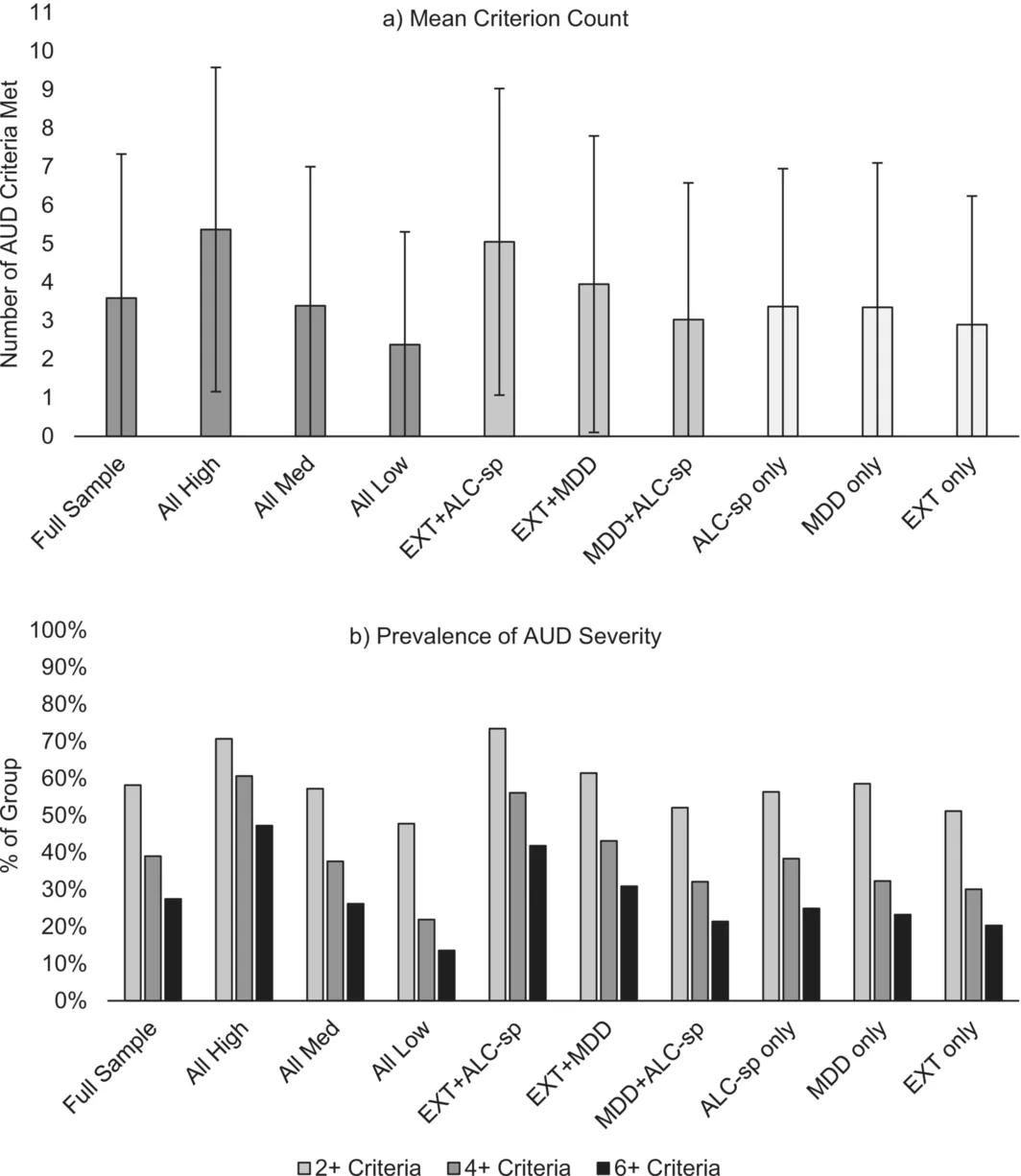
Metrics of DSM‐5 alcohol use disorder criteria (a) and disorder severity (b) by PGS classification group.

**TABLE 2:** Correlations between study variables in the full sample (*N* = 6883) and the polygenic score (PGS) classification group subsample (*N* = 2006).

	Domain	PGS			Criterion Count				AEQ				Traits	
	Variable	EXT	MDD	ALC‐sp	AUD	CD	ASPD	MDD	Pleasure	Arousal	Assert	Relax	Sen. Seek.	Neur.
PGS	EXT	—	0.47***	−0.09***	0.19***	0.15***	0.23***	0.17**	0.01	0.08**	0.05	0.05	0.18	0.11***
	MDD	0.30***	—	0.02	0.12***	0.14***	0.15***	0.15**	−0.01	0.06*	0.06	0.05	0.09	0.18***
	ALC‐sp	−0.07***	0.04**	—	0.14***	0.00	0.05*	0.03	0.03	0.08**	0.07*	0.05*	0.08	0.05*
Criterion count	AUD	0.19***	0.10***	0.12***	—	0.31***	0.55***	0.26***	0.26***	0.41***	0.35***	0.33***	0.24***	0.29***
	CD	0.15***	0.11***	0.02	0.31***	—	0.60***	0.24***	0.15***	0.23***	0.20***	0.22***	0.44***	0.24***
	ASPD	0.21***	0.12***	0.04**	0.55***	0.58***	—	0.23***	0.19***	0.31***	0.25***	0.29***	0.46***	0.30***
	MDD	0.09**	0.13***	0.03	0.28***	0.22***	0.24***	—	0.04	0.18**	0.14*	0.13*	−0.02	0.40***
AEQ	Pleasure	0.03*	−0.01	0.04**	0.29***	0.17***	0.20***	0.10**	—	0.69***	0.76***	0.77***	0.15	0.19***
	Arousal	0.08***	0.05**	0.07***	0.40***	0.21***	0.28***	0.20***	0.68***	—	0.84***	0.76***	0.29**	0.35***
	Assert.	0.05**	0.03*	0.06***	0.34***	0.17***	0.23***	0.16***	0.76***	0.84***	—	0.78***	0.26*	0.34***
	Relax	0.05**	0.04*	0.05**	0.33***	0.21***	0.26***	0.14***	0.76***	0.76***	0.78***	—	0.29**	0.28***
Traits	Sen. Seek.	0.21***	0.01	0.01	0.25***	0.32***	0.37***	−0.04	0.17***	0.21***	0.10	0.28***	—	−0.03
	Neur.	0.09***	0.14***	0.04**	0.28***	0.21***	0.27***	0.36***	0.20***	0.34***	0.32***	0.27***	0.00	—

*Note:* Correlations for full sample below the diagonal; correlations for nine‐group subsample above the diagonal.

Abbreviations: AEQ, Alcohol Expectancy Questionnaire; ALC‐sp, alcohol‐specific PGS; ASPD, antisocial personality disorder; AUD, alcohol use disorder; CD, conduct disorder; EXT, externalizing PGS; MDD, major depressive disorder; PGS were standardized.

*** *p* < 0.0001.

** *p* < 0.005.

* *p* < 0.05.

### 3.2 Alcohol Use Disorder Outcomes

Group descriptives, AUD criterion count, and AUD diagnosis prevalence by PGS classification group are presented in Table 3. Of the full sample, 2006 individuals fell into one of the nine PGS classification groups of interest. Pairwise comparisons for number of AUD criteria met across the nine PGS classification groups are depicted in Figure 2 (detailed results of all 36 pairwise comparisons are available in Table S1). The omnibus difference across PGS classification groups was significant (*χ* ^2^(8) = 79.15, *p* < 0.0001). In line with hypotheses, individuals high on all three PGS met more criteria (M = 5.37) compared to all other groups (M = 2.90–3.95) except EXT + ALC‐sp (M = 5.05), which also had a higher mean AUD criterion count than nearly all other groups. Groups with two high PGS variably met more AUD criteria (M = 3.03–5.05) than groups with one high PGS (M = 2.90–3.37) or all low PGS (M = 2.38), except the MDD + ALC‐sp group, which did not significantly differ from any of the groups with 1 high or all low PGS. This pattern suggests that EXT liability confers more risk, especially in conjunction with other genetic risk, than MDD liability. In contrast to hypotheses that EXT only and ALC‐sp only groups would meet more AUD criteria than the MDD only group, these groups did not significantly differ from each other, though these groups all had higher AUD criterion counts than the low PGS group. Overall, the largest effects were observed for the all high and EXT + ALC‐sp groups compared to the other groups. Results from analyses predicting AUD criterion count from continuous PGS and their interactions are presented in Table S2.

**TABLE 3:** Metrics of DSM‐5 alcohol use disorder outcomes by polygenic score (PGS) classification group.

EXT	MDD	ALC‐sp	# High PGS	# Low PGS	*N*	Female	Mean criterion count (SD)	2+ Criteria	4+ Criteria	6+ Criteria
High	High	High	3	0	239	48.95%	5.37 (4.21)	70.71%	60.66%	47.27%
High	High	Low	2	1	262	51.53%	3.95 (3.85)	61.45%	43.14%	30.92%
High	Low	High	2	1	98	56.12%	5.05 (3.98)	73.47%	56.11%	41.83%
Low	High	High	2	1	140	55.00%	3.03 (3.55)	52.14%	32.13%	21.42%
High	Low	Low	1	2	123	53.66%	2.90 (3.34)	51.22%	30.10%	20.34%
Low	High	Low	1	2	99	50.51%	3.35 (3.75)	58.59%	32.32%	23.23%
Low	Low	High	1	2	305	46.89%	3.37 (3.58)	56.39%	38.36%	24.92%
Low	Low	Low	0	3	251	54.18%	2.38 (2.93)	47.81%	21.93%	13.56%
Med	Med	Med	0	0	489	52.56%	3.39 (3.61)	57.26%	37.63%	26.18%
Full subsample			Range = 0–3		2006	51.65%	3.59 (3.74)	58.23%	39.03%	27.47%

*Note:* ALC‐sp: alcohol‐specific PGS; EXT, externalizing PGS; MDD, major depressive disorder PGS; mild or higher: 2+ criteria met; moderate or higher: 4+ criteria met; severe: 6+ criteria met.

**FIGURE 2:**
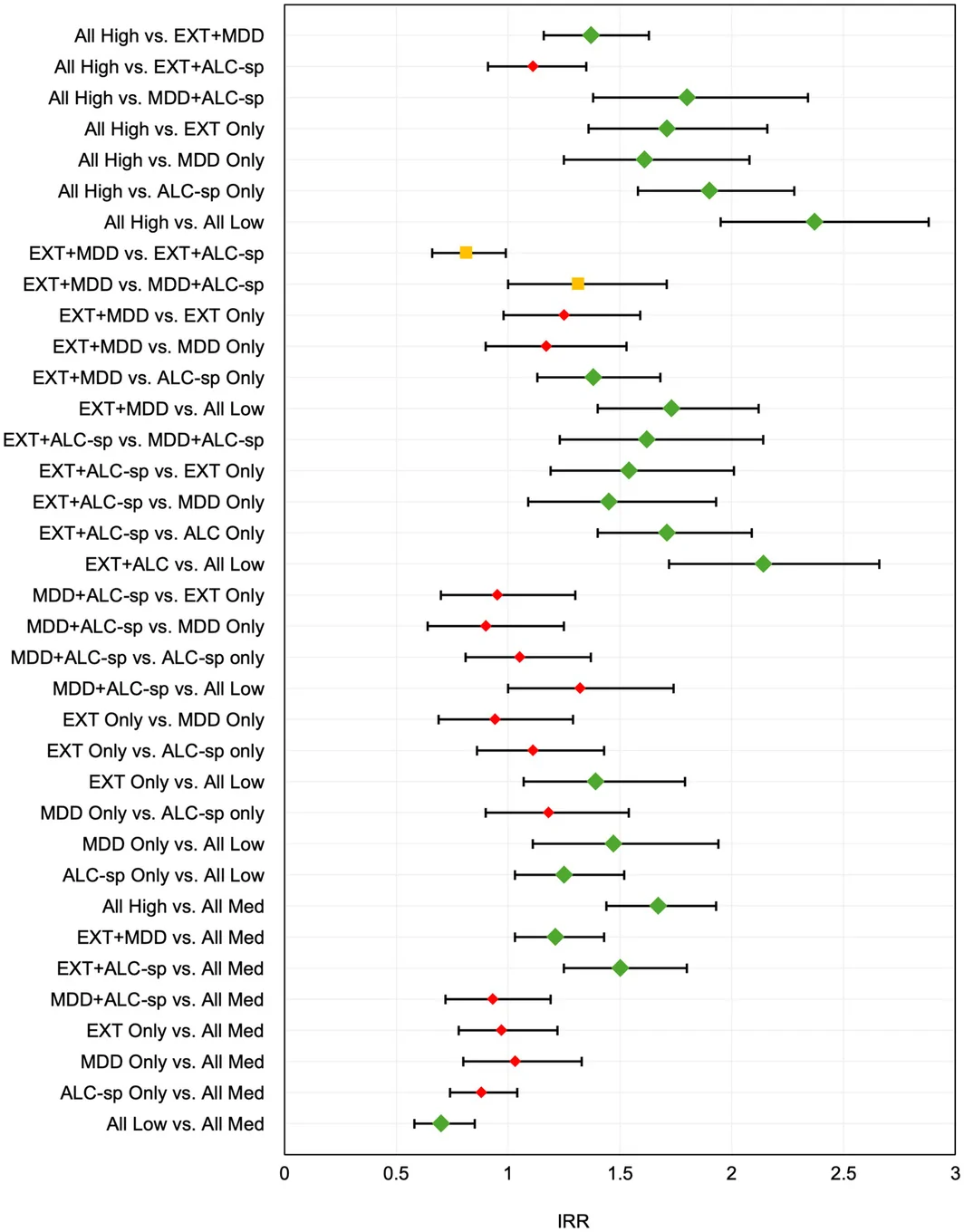
Incidence rate ratios (IRRs) of pairwise comparisons of DSM‐5 alcohol use disorder criteria across 9 PGS classification groups. Green circles denote statistical significance; yellow squares denote statistical significance before, but not after, FDR correction; red diamonds denote nonsignificance.

Pairwise comparisons for AUD diagnosis (moderate or severe; 4+ criterion threshold) across the nine PGS classification groups are depicted in Figure 3 (detailed results of all 36 pairwise comparisons are available in Table S3). The omnibus difference across PGS classification groups was significant (*χ* ^2^(8) = 81.73, *p* < 0.0001). Patterns of effect were almost entirely consistent with the results for the AUD criterion count outcome. Pairwise comparisons for any AUD severity (2+ criterion threshold) across the nine PGS classification groups are presented in the Supporting Information (Table S4, Figure S1), as are results from analyses predicting varying thresholds of AUD diagnosis from continuous PGS and their interactions (Table S5).

**FIGURE 3:**
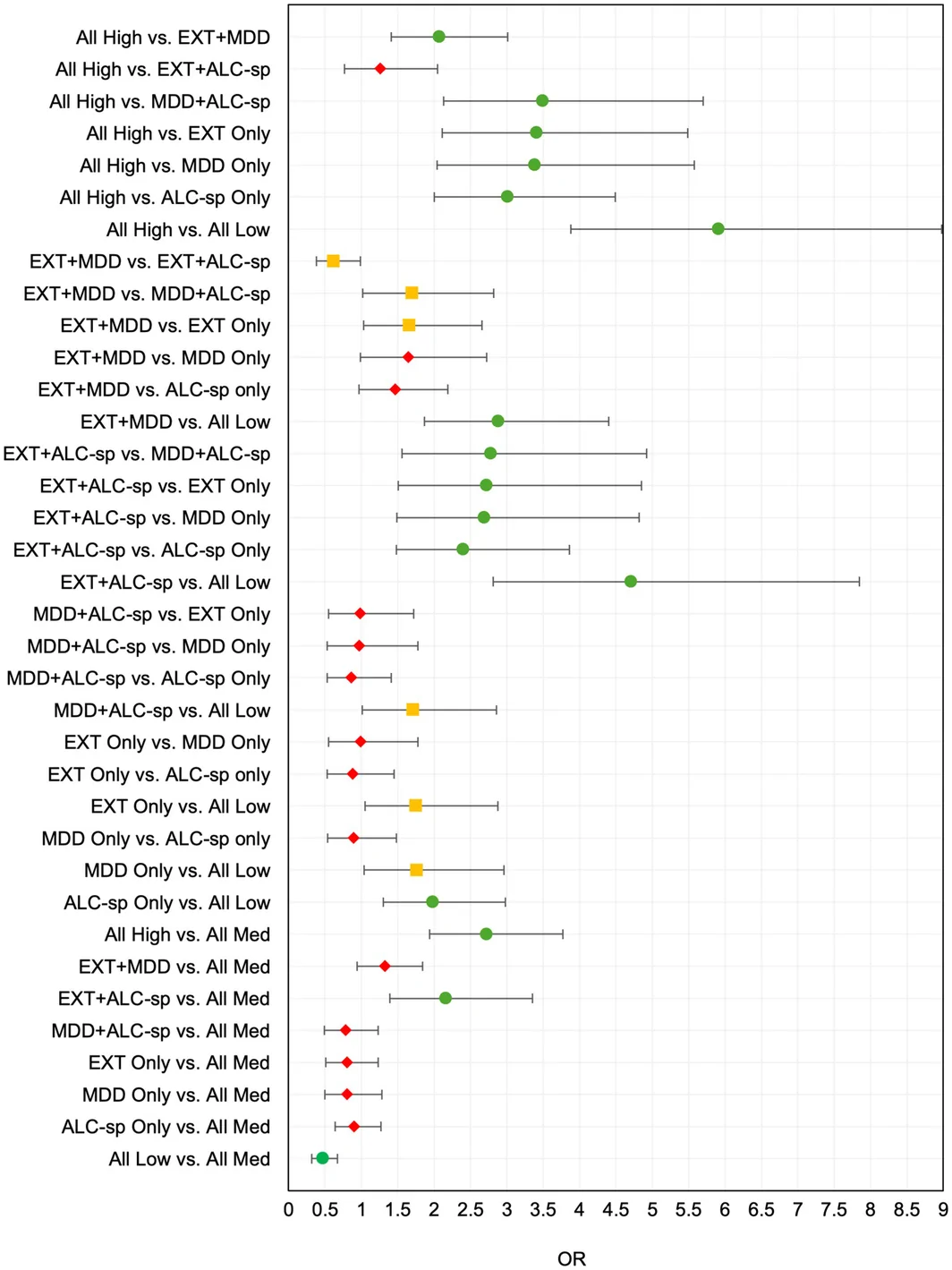
Odds ratios (ORs) of pairwise comparisons of DSM‐5 moderate to severe alcohol use disorder diagnosis (4+ criteria) across nine PGS classification groups. Green circles denote statistical significance; yellow squares denote statistical significance before, but not after, FDR correction; red diamonds denote nonsignificance.

### 3.3 Psychopathology, Alcohol Expectancies, and Personality Traits in the AUD Subsample

Results of the AUD subsample analyses comparing individuals with (1) high versus low EXT PGS, (2) high versus low MDD PGS, and (3) high versus low ALC‐sp PGS are presented in Table 4. Across all three PGS, having a high (vs. low) PGS was associated with having a higher AUD criterion count. Those with high (vs. low) EXT PGS had higher rates of CD (23.30% vs. 14.86%), more CD criteria met (M = 1.54 vs. 1.18), higher rates of ASPD (24.09% vs. 14.99%), more ASPD criteria met (M = 3.29 vs. 2.65), and slightly higher arousal/aggression (M = 30.52 vs. 29.75) and relaxation/tension reduction alcohol expectancies (M = 31.27 vs. 30.29). Those with high (vs. low) MDD PGS had higher rates of MDD (31.97% vs. 17.60%), more MDD criteria met (M = 6.16 vs. 5.54), and higher levels of neuroticism (M = 58.82 vs. 54.39). There were no differences in alcohol expectancies. Those with high (vs. low) ALC‐sp PGS had slightly higher arousal/aggression alcohol expectancies (M = 30.27 vs. 29.57); no other differences emerged. Results for the same analyses conducted in the subsample with any AUD severity (2+ criterion threshold) are presented in the Supporting Information (Table S6), as are results from analyses predicting psychopathology, alcohol expectancies, and personality traits from continuous PGS and their interactions in the AUD subsample among individuals with any severity AUD (2+ criterion threshold) and moderate or severe AUD (4+ criterion threshold; Tables S7–S10).

**TABLE 4:** Pairwise comparisons among individuals with DSM‐5 moderate or severe alcohol use disorder across levels of externalizing (EXT), major depressive disorder (MDD), and alcohol‐specific (ALC‐sp) polygenic scores (PGS).

Outcomes	EXT PGS		Difference test	MDD PGS		Difference test	ALC‐sp PGS		Difference test
	High (*N* = 1032)	Low (*N* = 632)	High vs. Low	High (*N* = 910)	Low (*N* = 710)	High vs. Low	High (*N* = 962)	Low (*N* = 707)	High vs. Low
Psychopathology (lifetime diagnosis)	%	%	OR (95% CI)	%	%	OR (95% CI)	%	%	OR (95% CI)
DSM‐IV CD	23.30%	14.86%	1.88 (1.40, 2.53)***	21.94%	14.59%	1.92 (1.40, 2.63)***	17.26%	20.66%	0.86 (0.64, 1.16)
DSM‐IV ASPD	24.09%	14.99%	1.99 (1.50, 2.65)***	21.66%	15.59%	1.86 (1.37, 2.52)***	19.65%	19.45%	1.01 (0.96, 1.07)
DSM‐IV MDD	27.63%	24.45%	1.15 (0.86, 1.52)	31.97%	17.60%	2.09 (1.54, 2.84)***	25.93%	29.07%	0.88 (0.68, 1.15)

Criterion count (lifetime maximum)	M (SD)	M (SD)	IRR (95% CI)	M (SD)	M (SD)	IRR (95% CI)	M (SD)	M (SD)	IRR (95% CI)
DSM‐5 AUD	7.73 (2.60)	7.20 (2.58)	1.09 (1.05, 1.13)***	7.83 (2.60)	7.29 (2.57)	1.09 (1.05, 1.13)***	7.81 (2.60)	7.29 (2.53)	1.07 (1.03, 1.10)**
DSM‐IV CD	1.54 (1.70)	1.18 (1.47)	1.31 (1.16, 1.49)***	1.48 (1.65)	1.17 (1.39)	1.36 (1.20, 1.54)***	1.31 (1.52)	1.39 (1.54)	0.98 (0.87, 1.11)
DSM‐IV ASPD	3.29 (1.76)	2.65 (1.65)	1.28 (1.21, 1.35)***	3.18 (1.70)	2.87 (1.70)	1.16 (1.09, 1.23)***	3.05 (1.76)	3.10 (1.72)	1.08 (0.82, 1.43)
DSM‐IV MDD	5.22 (3.74)	4.50 (3.77)	1.27 (1.01, 1.59)*	6.16 (3.32)	4.54 (3.55)	1.38 (1.15, 1.65)**	4.84 (3.85)	5.27 (3.64)	0.87 (0.69, 1.09)

Alcohol expectancies	M (SD)	M (SD)	B (95% CI)	M (SD)	M (SD)	B (95% CI)	M (SD)	M (SD)	B (95% CI)
Phys/Soc pleasure	32.88 (7.44)	32.89 (7.03)	−0.09 (−0.90, 0.71)	32.51 (7.53)	33.41 (6.93)	−0.46 (−1.31, 0.38)	32.76 (7.24)	32.73 (7.35)	0.18 (−0.66, 1.01)
Arousal/Aggression	30.52 (7.31)	29.75 (7.00)	0.83 (0.01, 1.66)*	29.95 (7.45)	29.99 (6.98)	−0.13 (−0.68, 0.94)	30.27 (7.10)	29.57 (7.16)	0.83 (0.01, 1.66)*
Social assertiveness	36.25 (9.38)	35.23 (9.33)	1.02 (−0.07, 2.12)	35.59 (9.67)	35.67 (9.00)	0.33 (−0.76, 1.41)	35.84 (9.35)	35.27 (9.31)	0.66 (−0.40, 1.72)
Relaxation	31.27 (7.60)	30.29 (7.72)	0.89 (0.00, 1.79)*	30.65 (7.87)	30.58 (7.48)	0.52 (−0.35, 1.39)	30.86 (7.71)	30.35 (7.61)	0.61 (−0.28, 1.51)

Personality	M (SD)	M (SD)	B (95% CI)	M (SD)	M (SD)	B (95% CI)	M (SD)	M (SD)	B (95% CI)
Sensation seeking	17.96 (4.06)	16.64 (5.37)	0.28 (−1.99, 2.56)	17.64 (3.99)	17.59 (4.33)	0.51 (−1.25, 2.27)	17.51 (4.52)	17.08 (3.92)	0.29 (−1.59, 2.17)
Neuroticism	56.14 (10.97)	55.34 (11.08)	0.52 (−0.81, 1.84)	56.82 (11.55)	54.39 (11.09)	2.32 (0.98, 3.68)**	56.10 (11.39)	56.31 (10.95)	0.05 (−1.30, 1.41)

*Note:* ASPD, antisocial personality disorder; AUD, alcohol use disorder; CD, conduct disorder; IRR, incidence rate ratio; MDD, major depressive disorder; OR, odds ratio; “low” used as reference category; difference tests derived from models that include age, sex, and ancestry principal components as covariates.

*** *p* < 0.0001.

** *p* < 0.001.

* *p* < 0.05.

## 4 Discussion

The present study examined the joint influences of polygenic risk for externalizing (EXT), internalizing (INT), and alcohol‐specific effects (ALC‐sp) on AUD outcomes. Participants with high EXT PGS and high ALC‐sp PGS, but low MDD PGS, had comparable AUD outcomes to participants with high levels of all three PGS. High MDD PGS generally did not confer additional risk for AUD when coupled with high EXT and/or ALC‐sp PGS. Somewhat surprisingly, AUD outcomes did not differ across participants with only one high PGS (i.e., EXT only, MDD only, ALC‐sp only). In other words, specificity in type of genetic risk appears to become increasingly salient as quantity of genetic risk factors increases. Previous studies have found that a substantial portion of the variance shared between internalizing and AUD is accounted for by variance shared with externalizing; this has been attributed to the distress and negative emotionality that can characterize both externalizing and internalizing pathways (Brasher et al. 2024; Foster et al. 2018). This may account for the observed influence of MDD in the absence of high EXT or ALC‐sp, but lack of additive MDD PGS influence in the presence of high EXT or ALC‐sp PGS. These patterns underscore that both quantity and source/type of genetic risk are salient in shaping AUD outcomes.

With respect to comorbid psychiatric disorders, alcohol expectancies, and personality traits among individuals with AUD with differing genetic risk profiles, patterns of association were largely aligned with hypotheses. High EXT and MDD PGS showed expected associations with CD and MDD, respectively, as well as personality traits, providing proof‐of‐concept regarding these pathways of risk. EXT PGS was hypothesized to underlie physical/social pleasure and arousal/aggression expectancies due to their more overtly “externalizing” nature (e.g., drinking driven by sensation seeking, propensity for aggression/antagonism); similarly, MDD PGS was expected to underlie social assertiveness and relaxation/tension reduction due to their more overtly “internalizing” nature (e.g., drinking to reduce fear/withdrawal, distress/negative emotionality). However, individuals with high EXT PGS most strongly endorsed alcohol expectancies, without substantive differentiation across expectancy types. Rather than specificity in associations with various alcohol expectancies, high EXT PGS may broadly potentiate alcohol expectancies that reinforce harmful patterns of use among people with AUD. MDD PGS was either unassociated or negatively associated with expectancies, supporting findings that some dimensions of internalizing (e.g., fear/withdrawal) can be protective against problematic drinking (Foster et al. 2018). High ALC‐sp PGS was uniquely associated with arousal/aggression, but not other alcohol expectancies. This is consistent with existing literature that has shown an AUD PGS to be associated with alcohol‐related aggression (Spychala et al. 2024). Individuals who metabolize alcohol more rapidly often experience more stimulating effects of alcohol, one mechanism by which high ALC‐sp PGS may be associated with arousal/aggression expectancies (Boyd and Corbin 2018). In sum, these findings shed light on a complex relationship between these genetically influenced pathways of risk for AUD and related phenotypes, including the dominant role of externalizing liability. Given the potentially unique influence of particular facets of externalizing (e.g., aggression) and internalizing (e.g., fear/withdrawal) on AUD outcomes, future work should evaluate how polygenic risk for distinct dimensions of each of these pathways may contribute to risk factors for AUD and, in turn, development of AUD (Kinstler et al. 2025).

With the field of psychiatric genetics at the precipice of translating PGS for substance use disorders to clinical application (Chen et al. 2023; Dick et al. 2025, 2026; Ramsey et al. 2021; Wray et al. 2021), the clinical implications for the results presented here are significant. PGS for externalizing and internalizing liability, and risk factors specific to certain forms of substance use, can be included in clinical risk prediction models (Barr et al. 2022; Dick et al. 2026). Other work by our team shows that the use of multiple PGS (e.g., EXT and ALC‐sp) improves prediction of substance use disorder outcomes (Poore et al. 2026), and these analyses suggest that the type of genetic risk one carries may also have implications for clinical presentation and comorbidities. Because genetic risk inherently precedes manifestation of behavioral disorders, a genetically informed approach offers prospective risk prediction free of temporal confounding and carries the advantage of understanding risk even before the onset of alcohol initiation or alcohol‐related problems (Dick et al. 2025, 2026). This has important implications for prevention and intervention. For example, use of personalized genetic feedback for substance use disorder risk is being piloted and evaluated in both community and clinical settings (Dash et al. 2026; Dick et al. 2025). This approach currently provides PGS‐based personalized genetic feedback on relative risk for developing a substance use disorder, grounded primarily in broad risk for externalizing (Dick et al. 2025; Karlsson Linnér et al. 2021), in combination with other behavioral and environmental risk factors. Findings from the present study indicate there may be utility in expanding genetic feedback to include multiple pathways of risk, particularly given that initial results show high levels of internalizing characteristics in clinical pilot samples (Poore et al. n.d.).

## 5 Limitations

Several study limitations should be considered. First, our analyses focused on a sample enriched for AUD. As such, results may not be generalizable to the general population. Relatedly, the study included only individuals of European‐like ancestry because available discovery GWAS were conducted exclusively in samples of individuals of European‐like ancestry, and there are known issues with the portability of GWAS to other ancestral groups (Lupi et al. 2024). As such, results may not be generalizable to individuals of non‐European ancestry. Second, at the time analyses were run, there was no multivariate GWAS of internalizing disorders from which an internalizing PGS comparable to the EXT PGS could be constructed. The EXT PGS uses summary statistics from a multivariate GWAS and captures shared genetic variance across a range of externalizing indicators including general risk tolerance, ADHD, risky sex, smoking, cannabis use, and alcohol problems and is thought to represent a liability toward behavioral undercontrol (Karlsson Linnér et al. 2021; Poore et al. 2023). A comparable approach to capture multiple dimensions of internalizing may provide more nuanced insight into the topics addressed here. Additionally, the ALC‐sp PGS represents the residual from the analysis with EXT, so it includes any genetic effects that impact alcohol problems that do not operate through the externalizing PGS. Finally, we did not evaluate sex differences in the effects of the three PGS on AUD and other outcomes as this was outside the scope of the present study but provides a fruitful avenue for future research in this area.

## 6 Conclusions

The findings presented here build upon a broad foundation of research from the twin and developmental literatures, extending findings to elucidate how polygenic risk for externalizing, internalizing, and response to alcohol act in concert to influence AUD outcomes. Internalizing symptoms are often less predictive of AUD, likely driven by lack of differentiation between specific dimensions of internalizing, some of which serve a protective effect against AUD (e.g., fear, withdrawal), while others overlap with features of externalizing and increase risk for AUD (e.g., distress, negative emotionality) (Foster et al. 2018). Findings from the present study support this, demonstrating that genetic influences on externalizing, internalizing, and response to alcohol operate at different magnitudes and in different ways to shape risk for AUD. While many studies continue to evaluate the effects of these PGS in isolation, it will be important to simultaneously account for these multiple pathways of genetic risk as research continues to refine etiologic frameworks of AUD.

## Funding

This work was supported by the National Institute on Alcohol Abuse and Alcoholism (U10AA008401, P50AA022537, K01AA031724) and the National Institute on Drug Abuse (R01DA050721, T32DA55569).

## Conflicts of Interest

D.M.D. is the cofounder and Chief Scientific Officer of Thrive Genetics Inc. and is also on the Advisory Board for the Seek Women's Health Company. She owns stock in both companies. She received royalties from authoring the book, The Child Code: Understanding Your Child's Unique Nature for Happier, More Effective Parenting, published by Avery, an imprint of the Penguin group.

## Supporting information


**Figure S1:** Odds ratios (ORs) of pairwise comparisons of DSM‐5 alcohol use disorder diagnosis (2+ criteria threshold) across nine Polygenic scores (PGS) classification groups.
**Table S1:** Pairwise comparisons across nine PGS classification groups for DSM‐5 alcohol use disorder criterion count.
**Table S2:** Models predicting DSM‐5 alcohol use disorder criterion count from EXT, MDD, and ALC‐sp PGS.
**Table S3:** Pairwise comparisons across nine PGS classification groups for DSM‐5 moderate to severe alcohol use disorder diagnosis (4+ criteria).
**Table S4:** Pairwise comparisons across nine PGS classification groups for any severity DSM‐5 alcohol use disorder diagnosis (2+ criteria).
**Table S5:** Models predicting varying thresholds of DSM‐5 alcohol use disorder diagnosis from EXT, MDD, and ALC‐sp PGS.
**Table S6:** Pairwise comparisons among individuals with any severity DSM‐5 alcohol use disorder (2+ criteria) across levels of EXT PGS, MDD, and ALC‐sp PGS.
**Table S7:** Models predicting externalizing outcomes from combinations of EXT and MDD PGS among individuals with any severity DSM‐5 alcohol use disorder (2+ criteria).
**Table S8:** Models predicting externalizing outcomes from combinations of EXT and MDD PGS among individuals with moderate to severe DSM‐5 alcohol use disorder (4+ criteria).
**Table S9:** Models predicting internalizing outcomes from combinations of EXT and MDD PGS among individuals with any severity DSM‐5 alcohol use disorder (2+ criteria).
**Table S10:** Models predicting internalizing outcomes from combinations of EXT and MDD PGS among individuals with DSM‐5 moderate to severe alcohol use disorder (4+ criteria).

## Data Availability

The data that support the findings of this study are available on request from the corresponding author. The data are not publicly available due to privacy or ethical restrictions.

## References

[acer70390-bib-0001] Adams, M. J. , F. Streit , X. Meng , et al. 2025. “Trans‐Ancestry Genome‐Wide Study of Depression Identifies 697 Associations Implicating Cell Types and Pharmacotherapies.” Cell 188: 640–652.e649. 10.1016/j.cell.2024.12.002 PMC11829167 39814019

[acer70390-bib-0002] Agrawal, A. , S. J. Brislin , K. K. Bucholz , et al. 2023. “The Collaborative Study on the Genetics of Alcoholism: Overview.” Genes, Brain, and Behavior 22: e12864. 10.1111/gbb.12864 PMC10550790 37736010

[acer70390-bib-0003] Barr, P. B. , M. N. Driver , S. I. Kuo , et al. 2022. “Clinical, Environmental, and Genetic Risk Factors for Substance Use Disorders: Characterizing Combined Effects Across Multiple Cohorts.” Molecular Psychiatry 27: 4633–4641. 10.1038/s41380-022-01801-6 PMC9938102 36195638

[acer70390-bib-0004] Benjamini, Y. , and Y. Hochberg . 1995. “Controlling the False Discovery Rate: A Practical and Powerful Approach to Multiple Testing.” Journal of the Royal Statistical Society, Series B: Statistical Methodology 57: 289–300.

[acer70390-bib-0005] Boyd, S. J. , and W. R. Corbin . 2018. “Faster Alcohol Metabolism Is Associated With Increased Stimulation and Within Session Consumption.” Experimental and Clinical Psychopharmacology 26: 168–176. 10.1037/pha0000176 29355349

[acer70390-bib-0006] Brasher, M. S. , A. D. Grotzinger , N. P. Friedman , H. R. Smolker , and L. M. Evans . 2024. “Disentangling Differing Relationships Between Internalizing Disorders and Alcohol Use.” American Journal of Medical Genetics Part B: Neuropsychiatric Genetics 195: e32975. 10.1002/ajmg.b.32975 PMC11147714 38375614

[acer70390-bib-0007] Brown, S. A. , B. A. Christiansen , and M. S. Goldman . 1987. “The Alcohol Expectancy Questionnaire: An Instrument for the Assessment of Adolescent and Adult Alcohol Expectancies.” Journal of Studies on Alcohol 48: 483–491. 10.15288/jsa.1987.48.483 3669677

[acer70390-bib-0008] Bucholz, K. K. , R. Cadoret , C. R. Cloninger , et al. 1994. “A New, Semi‐Structured Psychiatric Interview for Use in Genetic Linkage Studies: A Report on the Reliability of the SSAGA.” Journal of Studies on Alcohol 55: 149–158. 10.15288/jsa.1994.55.149 8189735

[acer70390-bib-0009] Chang, C. C. , C. C. Chow , L. C. Tellier , S. Vattikuti , S. M. Purcell , and J. J. Lee . 2015. “Second‐Generation PLINK: Rising to the Challenge of Larger and Richer Datasets.” GigaScience 4: s13742‐13015‐10047‐13748. 10.1186/s13742-015-0047-8 PMC4342193 25722852

[acer70390-bib-0010] Chen, L.‐S. , T. B. Baker , A. Ramsey , C. I. Amos , and L. J. Bierut . 2023. “Genomic Medicine to Reduce Tobacco and Related Disorders: Translation to Precision Prevention and Treatment.” Addiction Neuroscience 7: 100083. 10.1016/j.addicn.2023.100083 PMC10434839 37602286

[acer70390-bib-0011] Choi, S. W. , T. S.‐H. Mak , and P. F. O'Reilly . 2020. “Tutorial: A Guide to Performing Polygenic Risk Score Analyses.” Nature Protocols 15: 2759–2772. 10.1038/s41596-020-0353-1 PMC7612115 32709988

[acer70390-bib-0012] Cooper, M. L. 1994. “Motivations for Alcohol Use Among Adolescents: Development and Validation of a Four‐Factor Model.” Psychological Assessment 6: 117–128.

[acer70390-bib-0013] Costa, P. T. , and R. R. McCrae . 1992. Professional Manual: Revised NEO Personality Inventory (NEO‐PI‐R) and NEO Five‐Factor Inventory (NEO‐FFI). Psychological Assessment Resources.

[acer70390-bib-0014] Dash, G. F. , E. Balcke , H. Poore , and D. M. Dick . 2026. “Stakeholder Views on Implementing a Novel Addiction Screening and Prevention Tool in a Hospital Setting: A Qualitative Study.” Preprint. *medRxiv*. 10.64898/2026.04.14.26350880. 42665053

[acer70390-bib-0015] Deak, J. D. , and E. C. Johnson . 2021. “Genetics of Substance Use Disorders: A Review.” Psychological Medicine 51: 2189–2200. 10.1017/S0033291721000969 PMC8477224 33879270

[acer70390-bib-0016] Dick, D. M. , F. Aliev , S. J. Latendresse , et al. 2013. “Adolescent Alcohol Use Is Predicted by Childhood Temperament Factors Before Age 5, With Mediation Through Personality and Peers.” Alcoholism, Clinical and Experimental Research 37: 2108–2117. 10.1111/acer.12206 PMC3823677 23841856

[acer70390-bib-0017] Dick, D. M. , E. Balcke , V. McCutcheon , et al. 2023. “The Collaborative Study on the Genetics of Alcoholism: Sample and Clinical Data.” Genes, Brain, and Behavior 22: e12860. 10.1111/gbb.12860 PMC10550787 37581339

[acer70390-bib-0018] Dick, D. M. , M. Choi , E. Balcke , et al. 2025. “Development of the Comprehensive Addiction Risk Evaluation System (CARES): Initial Participant Response to an Online Personalized Feedback Program Integrating Genomic, Behavioral, and Environmental Risk Information.” Complex Psychiatry 11: 113–130. 10.1159/000547783 PMC12453580 40988992

[acer70390-bib-0019] Dick, D. M. , G. F. Dash , I.‐T. Hung , and A. Horgan . 2026. “Are Polygenic Scores for Psychiatric and Substance Use Outcomes “Ready” for Clinical Application? Current State and Next Steps.” Psychiatric Genetics 36: 57–68. 10.1097/YPG.0000000000000415 PMC13124273 41994983

[acer70390-bib-0020] Foster, K. T. , B. M. Hicks , and R. A. Zucker . 2018. “Positive and Negative Effects of Internalizing on Alcohol Use Problems From Childhood to Young Adulthood: The Mediating and Suppressing Role of Externalizing.” Journal of Abnormal Psychology 127: 394–403. 10.1037/abn0000337 PMC6849508 29745704

[acer70390-bib-0021] García, M. C. , L. M. Rossen , K. Matthews , et al. 2024. “Preventable Premature Deaths From the Five Leading Causes of Death in Nonmetropolitan and Metropolitan Counties, United States, 2010–2022.” MMWR Surveillance Summaries 73, no. 2: 1–11. 10.15585/mmwr.ss7302a1 PMC11065459 38687830

[acer70390-bib-0022] Ge, T. , C.‐Y. Chen , Y. Ni , Y.‐C. A. Feng , and J. W. Smoller . 2019. “Polygenic Prediction via Bayesian Regression and Continuous Shrinkage Priors.” Nature Communications 10: 1776. 10.1038/s41467-019-09718-5 PMC6467998 30992449

[acer70390-bib-0023] Hussong, A. M. , S. T. Ennett , M. J. Cox , and M. Haroon . 2017. “A Systematic Review of the Unique Prospective Association of Negative Affect Symptoms and Adolescent Substance Use Controlling for Externalizing Symptoms.” Psychology of Addictive Behaviors 31: 137–147. 10.1037/adb0000247 PMC5344716 28134539

[acer70390-bib-0024] Hussong, A. M. , D. J. Jones , G. L. Stein , D. H. Baucom , and S. Boeding . 2011. “An Internalizing Pathway to Alcohol Use and Disorder.” Psychology of Addictive Behaviors 25: 390–404. 10.1037/a0024519 PMC3178003 21823762

[acer70390-bib-0025] Johnson, E. C. , J. E. Salvatore , D. Lai , et al. 2023. “The Collaborative Study on the Genetics of Alcoholism: Genetics.” Genes, Brain, and Behavior 22: e12856. 10.1111/gbb.12856 PMC10550788 37387240

[acer70390-bib-0026] Karlsson Linnér, R. , T. T. Mallard , P. B. Barr , et al. 2021. “Multivariate Analysis of 1.5 Million People Identifies Genetic Associations With Traits Related to Self‐Regulation and Addiction.” Nature Neuroscience 24: 1367–1376. 10.1038/s41593-021-00908-3 PMC8484054 34446935

[acer70390-bib-0027] Kinstler, E. , A. J. Gorelik , S. E. Paul , et al. 2025. “Genetic Influences for Distinct Impulsivity Domains Are Differentially Associated With Early Substance Use Initiation: Results From the ABCD Study.” Psychological Medicine 55: e313. 10.1017/S0033291725101931 PMC12551585 41114439

[acer70390-bib-0028] LaBrie, J. W. , S. R. Kenney , L. E. Napper , and K. Miller . 2014. “Impulsivity and Alcohol‐Related Risk Among College Students: Examining Urgency, Sensation Seeking and the Moderating Influence of Beliefs About Alcohol's Role in the College Experience.” Addictive Behaviors 39: 159–164. 10.1016/j.addbeh.2013.09.018 PMC3864886 24120644

[acer70390-bib-0029] Levey, D. F. , M. B. Stein , F. R. Wendt , et al. 2021. “Bi‐Ancestral Depression GWAS in the Million Veteran Program and Meta‐Analysis in > 1.2 Million Individuals Highlight New Therapeutic Directions.” Nature Neuroscience 24: 954–963. 10.1038/s41593-021-00860-2 PMC8404304 34045744

[acer70390-bib-0030] Lupi, A. S. , A. I. Vazquez , and G. de Los Campos . 2024. “Mapping the Relative Accuracy of Cross‐Ancestry Prediction.” Nature Communications 15: 1–14. 10.1038/s41467-024-54727-8 PMC11612447 39622843

[acer70390-bib-0031] Martin, C. S. , D. L. Steinley , A. Vergés , and K. J. Sher . 2011. “The Proposed 2/11 Symptom Algorithm for DSM‐5 Substance‐Use Disorders Is Too Lenient.” Psychological Medicine 41: 2008–2010. 10.1017/S0033291711000717 PMC4245011 21557890

[acer70390-bib-0032] Poore, H. E. , E. Balcke , D. Patel , et al. n.d. Implementing the Comprehensive Addiction Risk Evaluation System (CARES) in a Clinical Setting: A Pilot Study.

[acer70390-bib-0033] Poore, H. E. , C. Chatzinakos , B. Leger , et al. 2026. “Multivariate Genetic Analyses of 2.2 Million Individuals Reveal Broad and Substance‐Specific Pathways of Addiction Risk.” Nature Mental Health 4: 582–593. 10.1038/s44220-026-00608-6 PMC13076202 41987933

[acer70390-bib-0034] Poore, H. E. , A. Hatoum , T. T. Mallard , et al. 2023. “A Multivariate Approach to Understanding the Genetic Overlap Between Externalizing Phenotypes and Substance Use Disorders.” Addiction Biology 28: e13319. 10.1111/adb.13319 PMC11010459 37644899

[acer70390-bib-0035] Ramsey, A. T. , J. L. Bourdon , M. Bray , et al. 2021. “Proof of Concept of a Personalized Genetic Risk Tool to Promote Smoking Cessation: High Acceptability and Reduced Cigarette Smoking.” Cancer Prevention Research (Philadelphia, Pa.) 14: 253–262. 10.1158/1940-6207.CAPR-20-0328 PMC7981287 32958583

[acer70390-bib-0036] Savage, J. E. , D. M. Dick , and Group SfSW . 2023. “Internalizing and Externalizing Subtypes of Alcohol Misuse and Their Relation to Drinking Motives.” Addictive Behaviors 136: 107461. 10.1016/j.addbeh.2022.107461 36063573

[acer70390-bib-0037] Schuckit, M. A. 2018. “A Critical Review of Methods and Results in the Search for Genetic Contributors to Alcohol Sensitivity.” Alcoholism: Clinical and Experimental Research 42: 822–835. 10.1111/acer.13628 PMC5916326 29623680

[acer70390-bib-0038] Schuckit, M. A. , T. L. Smith , G. P. Danko , et al. 2009. “An Evaluation of the Full Level of Response to Alcohol Model of Heavy Drinking and Problems in COGA Offspring.” Journal of Studies on Alcohol and Drugs 70: 436–445. 10.15288/jsad.2009.70.436 PMC2670749 19371495

[acer70390-bib-0039] Spychala, K. M. , E. W. Yeung , A. P. Miller , et al. 2024. “Genetic Risk for Trait Aggression and Alcohol Use Predict Unique Facets of Alcohol‐Related Aggression.” Psychology of Addictive Behaviors 31: 63–75. 10.1037/adb0001015 PMC11621227 38842867

[acer70390-bib-0040] Tarter, R. , L. Kirisci , A. Hegedus , A. Mezzich , and M. Vanyukov . 1994. “Heterogeneity of Adolescent Alcoholism.” Annals of the New York Academy of Sciences 708: 172–180. 10.1111/j.1749-6632.1994.tb24710.x 8154678

[acer70390-bib-0041] The International HapMap 3 Consortium . 2010. “Integrating Common and Rare Genetic Variation in Diverse Human Populations.” Nature 467: 52–58. 10.1038/nature09298 PMC3173859 20811451

[acer70390-bib-0042] Tully, E. C. , and W. G. Iacono . 2016. “An Integrative Common Liabilities Model for the Comorbidity of Substance Use Disorders With Externalizing and Internalizing Disorders.” In The Oxford Handbook of Substance Use and Substance Use Disorders, vol. 2, 187–214. Oxford University Press.

[acer70390-bib-0043] Wall, T. L. , S. E. Luczak , and S. Hiller‐Sturmhofel . 2016. “Biology, Genetics, and Environment: Underlying Factors Influencing Alcohol Metabolism.” Alcohol Research: Current Reviews 38: 59–68. 10.35946/arcr.v38.1.08 PMC4872614 27163368

[acer70390-bib-0044] World Health Organization . 2024. Global Status Report on Alcohol and Health and Treatment of Substance Use Disorders. World Health Organization. https://iris.who.int/handle/10665/377960.

[acer70390-bib-0045] Wray, N. R. , T. Lin , J. Austin , et al. 2021. “From Basic Science to Clinical Application of Polygenic Risk Scores: A Primer.” JAMA Psychiatry 78: 101–109. 10.1001/jamapsychiatry.2020.3049 32997097

[acer70390-bib-0046] Yeung, E. W. , L. Herchenroeder , H. Webster , A. B. Albright , and I. R. Gizer . 2024. “Genetic Influences on Alcohol Sensitivity: A Critical Review.” Current Addiction Reports 11: 94–104. 10.1007/s40429-023-00530-2 PMC12097646 40406603

[acer70390-bib-0047] Zhou, H. , R. Polimanti , B.‐Z. Yang , et al. 2017. “Genetic Risk Variants Associated With Comorbid Alcohol Dependence and Major Depression.” JAMA Psychiatry 74: 1234–1241. 10.1001/jamapsychiatry.2017.3275 PMC6331050 29071344

[acer70390-bib-0048] Zucker, R. A. 2008. “Anticipating Problem Alcohol Use Developmentally From Childhood Into Middle Adulthood: What Have We Learned?” Addiction (Abingdon, England) 103, no. Suppl 1: 100–108. 10.1111/j.1360-0443.2008.02179.x PMC2593849 18426543

[acer70390-bib-0049] Zuckerman, M. 2007. “The Sensation Seeking Scale V (SSS‐V): Still Reliable and Valid.” Personality and Individual Differences 43: 1303–1305.

[acer70390-bib-0050] Zuckerman, M. , E. A. Kolin , L. Price , and I. Zoob . 1964. “Development of a Sensation‐Seeking Scale.” Journal of Consulting Psychology 28: 477–482. 10.1037/h0040995 14242306

