# Supplementary material for: Polygenic Risk Profiles and the Manifestations of Alcohol Use Disorder and Related Outcomes
Source: Alcohol Clin Exp Res (Hoboken). 2026 Oct 12;50(10):e70390. doi: 10.1111/acer.70390 (PMC13657073; doi:10.1111/acer.70390)
Supplement: Supplementary file 1 [file ACER-50-0-s001.pdf]

Supplemental Materials

For

Polygenic Risk Profiles and the Manifestations of Alcohol Use Disorder and Related Outcomes

Genevieve F. Dash, Ph.D.,[1,2] Fazil Aliev, Ph.D.,[1,2] Sally I-Chun Kuo, Ph.D.,[1,2] Andrey Anokhin, Ph.D.,[3] Sarah J. Brislin, Ph.D.,[1,2] Kathleen Bucholz, Ph.D.,[3] Grace Chan, Ph.D.,[4, 7] Maia Choi, Ph.D.,[5] Emma C. Johnson, Ph.D.,[3] Chella Kamarajan, Ph.D.,[6] Samuel Kuperman, M.D.,[7] Alex P. Miller, Ph.D.,[8] John I. Nurnberger, M.D., Ph.D.,[8] Martin H. Plawecki, M.D.,[8] Holly E. Poore, Ph.D.,[1,2] John Rice, Ph.D.,[3] Leah Wetherill, Ph.D.,[9] COGA Collaborators, & Danielle M. Dick, Ph.D.[1,2]

[1] Department of Psychiatry, Rutgers Robert Wood Johnson Medical School, Piscataway, NJ

[2] Rutgers Addiction Research Center, Brain Health Institute, Piscataway, NJ

[3] Department of Psychiatry, WashU School of Medicine, St. Louis, MO

[4] Department of Psychiatry, UConn Health, Farmington, CT

[5] Department of Psychology, Rutgers University, New Brunswick, NJ

[6] Department of Psychiatry, SUNY Downstate Health Sciences University, Brooklyn, NY

[7] Department of Psychiatry, University of Iowa Carver College of Medicine, Iowa City, IA

[8] Department of Psychiatry, Indiana University School of Medicine, Indianapolis, IN

[9] Department of Medical and Molecular Genetics, Indiana University School of Medicine, Indianapolis, IN

Table S1

Pairwise comparisons across 9 PGS classification groups for DSM-5 alcohol use disorder criterion count

Corr.	PGS Group 1			PGS Group 2			Incidence	95% CI		Raw	FDR adj.	Sig before	Sig after
Hyp.	EXT	MDD	ALC-sp	EXT	MDD	ALC-sp	Rate Ratio	Lower	Upper	*p*-value	*p*-value	correction	correction
H1	High	High	High	High	High	Low	1.37	1.16	1.63	.0002	.0007	Y	Y
H1	High	High	High	High	Low	High	1.11	0.91	1.35	.2913	.3745	N	N
H1	High	High	High	Low	High	High	1.80	1.38	2.34	<.0001	<.0004	Y	Y
H1	High	High	High	High	Low	Low	1.71	1.36	2.16	<.0001	<.0004	Y	Y
H1	High	High	High	Low	High	Low	1.61	1.25	2.08	.0003	.0009	Y	Y
H1	High	High	High	Low	Low	High	1.90	1.58	2.28	<.0001	<.0004	Y	Y
H1	High	High	High	Low	Low	Low	2.37	1.95	2.88	<.0001	<.0004	Y	Y
-	High	High	Low	High	Low	High	0.81	0.66	0.99	.0352	.0603	Y	N
-	High	High	Low	Low	High	High	1.31	1.00	1.71	.0492	.0790	Y	N
H2	High	High	Low	High	Low	Low	1.25	0.98	1.59	.0763	.1145	N	N
H2	High	High	Low	Low	High	Low	1.17	0.90	1.53	.2435	.3247	N	N
H2	High	High	Low	Low	Low	High	1.38	1.13	1.68	.0013	.0031	Y	Y
H2	High	High	Low	Low	Low	Low	1.73	1.40	2.12	<.0001	<.0004	Y	Y
-	High	Low	High	Low	High	High	1.62	1.23	2.14	.0006	.0017	Y	Y
H2	High	Low	High	High	Low	Low	1.54	1.19	2.01	.0013	.0031	Y	Y
H2	High	Low	High	Low	High	Low	1.45	1.09	1.93	.0096	.0203	Y	Y
H2	High	Low	High	Low	Low	High	1.71	1.40	2.09	<.0001	<.0004	Y	Y
H2	High	Low	High	Low	Low	Low	2.14	1.72	2.66	<.0001	<.0004	Y	Y
H2	Low	High	High	High	Low	Low	0.95	0.70	1.30	.7516	.7958	N	N
H2	Low	High	High	Low	High	Low	0.90	0.64	1.25	.5110	.6132	N	N
H2	Low	High	High	Low	Low	High	1.05	0.81	1.37	.6977	.7689	N	N
H2	Low	High	High	Low	Low	Low	1.32	1.00	1.74	.0505	.0790	N	N
H4	High	Low	Low	Low	High	Low	0.94	0.69	1.29	.7048	.7689	N	N
-	High	Low	Low	Low	Low	High	1.11	0.86	1.43	.4285	.5319	N	N
H3	High	Low	Low	Low	Low	Low	1.39	1.07	1.79	.0120	.0240	Y	Y
H4	Low	High	Low	Low	Low	High	1.18	0.90	1.54	.2409	.3247	N	N
H3	Low	High	Low	Low	Low	Low	1.47	1.11	1.94	.0064	.0144	Y	Y
H3	Low	Low	High	Low	Low	Low	1.25	1.03	1.52	.0242	.0436	Y	Y
-	High	High	High	Med	Med	Med	1.67	1.44	1.93	<.0001	<.0004	Y	Y
-	High	High	Low	Med	Med	Med	1.21	1.03	1.43	.0220	.0417	Y	Y
-	High	Low	High	Med	Med	Med	1.50	1.25	1.80	<.0001	<.0004	Y	Y
-	Low	High	High	Med	Med	Med	0.93	0.72	1.19	.5434	.6310	N	N
-	High	Low	Low	Med	Med	Med	0.97	0.78	1.22	.8142	.8142	N	N
-	Low	High	Low	Med	Med	Med	1.03	0.80	1.33	.7955	.8142	N	N
-	Low	Low	High	Med	Med	Med	0.88	0.74	1.04	.1322	.1904	N	N
-	Low	Low	Low	Med	Med	Med	0.70	0.58	0.85	.0002	.0007	Y	Y

Note. Corr. Hyp=corresponding hypothesis.

Table S2

Models predicting DSM-5 alcohol use disorder criterion count from EXT, MDD, and ALC-sp PGS

Predictor	IRR	95% CI	Z	*p*
Model 1 (QIC=-28743.84; QICu=-28745.99)				
**Intercept**	**2.81**	**2.24, 3.52**	**8.96**	**<.0001**
**Age**	**0.99**	**0.99, 0.99**	**-9.27**	**<.0001**
**Sex**	**1.79**	**1.70, 1.88**	**21.96**	**<.0001**
**EXT PGS**	**1.23**	**1.20, 1.27**	**13.66**	**<.0001**
**MDD PGS**	**1.06**	**1.03, 1.10**	**3.99**	**<.0001**
**ALC-sp PGS**	**1.12**	**1.09, 1.16**	**7.72**	**<.0001**
Model 2 (QIC=-28744.92; QICu=-28747.60)				
**Intercept**	**2.80**	**2.23, 3.52**	**8.85**	**<.0001**
**Age**	**0.99**	**0.99, 0.99**	**-9.22**	**<.0001**
**Sex**	**1.79**	**1.70, 1.88**	**22.02**	**<.0001**
**EXT PGS**	**1.23**	**1.20, 1.27**	**13.82**	**<.0001**
**MDD PGS**	**1.06**	**1.03, 1.09**	**4.02**	**<.0001**
**ALC-sp PGS**	**1.13**	**1.09, 1.16**	**7.73**	**<.0001**
EXT PGS*MDD PGS	1.00	0.97, 1.02	-0.31	.76
EXT PGS*ALC-sp PGS	0.98	0.96, 1.01	-1.07	.28
MDD PGS*ALC-sp PGS	0.99	0.96, 1.02	-0.78	.44
Model 3 (QIC=-28750.85; QICu=-28753.31)				
**Intercept**	**2.80**	**2.23, 3.52**	**8.87**	**<.0001**
**Age**	**0.99**	**0.99, 0.99**	**-9.22**	**<.0001**
**Sex**	**1.79**	**1.70, 1.88**	**22.01**	**<.0001**
**EXT PGS**	**1.23**	**1.20, 1.26**	**13.80**	**<.0001**
**MDD PGS**	**1.06**	**1.03, 1.09**	**4.07**	**<.0001**
**ALC-sp PGS**	**1.12**	**1.08, 1.15**	**7.23**	**<.0001**
EXT PGS*MDD PGS	1.00	0.97, 1.02	-0.39	.69
EXT PGS*ALC-sp PGS	0.98	0.96, 1.01	-1.06	.28
MDD PGS*ALC-sp PGS	0.99	0.96, 1.02	-0.70	.48
EXT PGS*MDD PGS*ALC-sp PGS	1.02	0.99, 1.04	1.30	.19

Note. EXT PGS, MDD PGS, and ALC-sp PGS accounted for 3.5%, 0.00012%, and 0.4% of the variance in AUD criterion count, respectively; ancestry PCs were included as covariates in all models but are not reported here for ease of presentation; female used as reference category for sex; bold indicates statistical significance, $p<.05$.

Table S3

Pairwise comparisons across 9 PGS classification groups for DSM-5 moderate to severe alcohol use disorder diagnosis (4+ criteria)

Corr.	PGS Group 1			PGS Group 2			Odds Ratio	95% CI		Raw	FDR adj.	Sig before	Sig after
Hyp.	EXT	MDD	ALC-sp	EXT	MDD	ALC-sp		Lower	Upper	*p*-value	*p*-value	correction	correction
H1	High	High	High	High	High	Low	2.06	1.41	3.01	.0002	.0007	Y	Y
H1	High	High	High	High	Low	High	1.26	0.77	2.05	.3635	.4512	N	N
H1	High	High	High	Low	High	High	3.48	2.13	5.70	<.0001	<.0004	Y	Y
H1	High	High	High	High	Low	Low	3.40	2.11	5.49	<.0001	<.0004	Y	Y
H1	High	High	High	Low	High	Low	3.37	2.04	5.58	<.0001	<.0004	Y	Y
H1	High	High	High	Low	Low	High	3.00	2.00	4.49	<.0001	<.0004	Y	Y
H1	High	High	High	Low	Low	Low	5.90	3.88	8.98	<.0001	<.0004	Y	Y
-	High	High	Low	High	Low	High	0.61	0.38	0.99	.0459	.0781	Y	N
-	High	High	Low	Low	High	High	1.69	1.02	2.82	.0433	.0779	Y	N
H2	High	High	Low	High	Low	Low	1.65	1.03	2.66	.0384	.0728	Y	N
H2	High	High	Low	Low	High	Low	1.64	0.99	2.72	.0561	.0878	N	N
H2	High	High	Low	Low	Low	High	1.46	0.97	2.19	.0700	.1050	N	N
H2	High	High	Low	Low	Low	Low	2.87	1.87	4.40	<.0001	<.0004	Y	Y
-	High	Low	High	Low	High	High	2.77	1.56	4.92	.0005	.0015	Y	Y
H2	High	Low	High	High	Low	Low	2.71	1.51	4.85	.0008	.0021	Y	Y
H2	High	Low	High	Low	High	Low	2.68	1.49	4.82	.0010	.0024	Y	Y
H2	High	Low	High	Low	Low	High	2.39	1.48	3.86	.0004	.0013	Y	Y
H2	High	Low	High	Low	Low	Low	4.70	2.81	7.85	<.0001	<.0004	Y	Y
H2	Low	High	High	High	Low	Low	0.98	0.55	1.72	.9368	.9636	N	N
H2	Low	High	High	Low	High	Low	0.97	0.53	1.78	.9169	.9636	N	N
H2	Low	High	High	Low	Low	High	0.86	0.53	1.41	.5527	.6421	N	N
H2	Low	High	High	Low	Low	Low	1.70	1.01	2.86	.0477	.0781	Y	N
H4	High	Low	Low	Low	High	Low	0.99	0.55	1.78	.9743	.9743	N	N
-	High	Low	Low	Low	Low	High	0.88	0.53	1.45	.6208	.6984	N	N
H3	High	Low	Low	Low	Low	Low	1.74	1.05	2.88	.0331	.0701	Y	N
H4	Low	High	Low	Low	Low	High	0.89	0.54	1.48	.6527	.7120	N	N
H3	Low	High	Low	Low	Low	Low	1.75	1.04	2.96	.0355	.0710	Y	N
H3	Low	Low	High	Low	Low	Low	1.97	1.30	2.98	.0014	.0032	Y	Y
-	High	High	High	Med	Med	Med	2.71	1.94	3.77	<.0001	<.0004	Y	Y
-	High	High	Low	Med	Med	Med	1.32	0.94	1.84	.1119	.1611	N	N
-	High	Low	High	Med	Med	Med	2.15	1.39	3.35	.0006	.0017	Y	Y
-	Low	High	High	Med	Med	Med	0.78	0.49	1.23	.2810	.3891	N	N
-	High	Low	Low	Med	Med	Med	0.80	0.51	1.23	.3037	.4049	N	N
-	Low	High	Low	Med	Med	Med	0.80	0.50	1.28	.3561	.4512	N	N
-	Low	Low	High	Med	Med	Med	0.90	0.64	1.27	.5529	.6421	N	N
-	Low	Low	Low	Med	Med	Med	0.46	0.32	0.67	<.0001	<.0004	Y	Y

Note. Corr. Hyp=corresponding hypothesis.

Table S4

Pairwise comparisons across 9 PGS classification groups for any severity DSM-5 alcohol use disorder diagnosis (2+ criteria)

Corr.	PGS Group 1			PGS Group 2			Odds Ratio	95% CI		Raw	FDR adj.	Sig before	Sig after
Hyp.	EXT	MDD	ALC-sp	EXT	MDD	ALC-sp		Lower	Upper	*p*-value	*p*-value	correction	correction
H1	High	High	High	High	High	Low	1.51	1.00	2.30	.0510	.1102	N	N
H1	High	High	High	High	Low	High	0.87	0.49	1.54	.6304	.6877	N	N
H1	High	High	High	Low	High	High	2.34	1.41	3.88	.0009	.0054	Y	Y
H1	High	High	High	High	Low	Low	2.03	1.19	3.46	.0095	.0311	Y	Y
H1	High	High	High	Low	High	Low	1.68	0.99	2.84	.0551	.1102	N	N
H1	High	High	High	Low	Low	High	2.33	1.53	3.55	<.0001	<.0012	Y	Y
H1	High	High	High	Low	Low	Low	2.77	1.81	4.23	<.0001	<.0012	Y	Y
-	High	High	Low	High	Low	High	0.57	0.33	0.99	.0463	.1102	Y	N
-	High	High	Low	Low	High	High	1.55	0.96	2.50	.0736	.1395	N	N
H2	High	High	Low	High	Low	Low	1.34	0.82	2.18	.2395	.3593	N	N
H2	High	High	Low	Low	High	Low	1.11	0.67	1.82	.6901	.7307	N	N
H2	High	High	Low	Low	Low	High	1.54	1.03	2.29	.0333	.0922	Y	N
H2	High	High	Low	Low	Low	Low	1.83	1.22	2.73	.0032	.0128	Y	Y
-	High	Low	High	Low	High	High	2.70	1.48	4.91	.0011	.0057	Y	Y
H2	High	Low	High	High	Low	Low	2.33	1.25	4.37	.0081	.0292	Y	Y
H2	High	Low	High	Low	High	Low	1.93	1.02	3.67	.0444	.1102	Y	N
H2	High	Low	High	Low	Low	High	2.69	1.58	4.57	.0003	.0027	Y	Y
H2	High	Low	High	Low	Low	Low	3.19	1.89	5.39	<.0001	<.0012	Y	Y
H2	Low	High	High	High	Low	Low	0.87	0.49	1.52	.6127	.6877	N	N
H2	Low	High	High	Low	High	Low	0.72	0.40	1.27	.2497	.3596	N	N
H2	Low	High	High	Low	Low	High	1.00	0.64	1.56	.9857	.9857	N	N
H2	Low	High	High	Low	Low	Low	1.18	0.74	1.88	.4840	.6223	N	N
H4	High	Low	Low	Low	High	Low	0.83	0.45	1.51	.5357	.6650	N	N
-	High	Low	Low	Low	Low	High	1.15	0.70	1.90	.5799	.6877	N	N
H3	High	Low	Low	Low	Low	Low	1.37	0.84	2.23	.2133	.3339	N	N
H4	Low	High	Low	Low	Low	High	1.39	0.84	2.31	.2020	.3339	N	N
H3	Low	High	Low	Low	Low	Low	1.65	0.99	2.75	.0543	.1102	N	N
H3	Low	Low	High	Low	Low	Low	1.19	0.83	1.69	.3413	.4551	N	N
-	High	High	High	Med	Med	Med	1.90	1.33	2.72	.0005	.0036	Y	Y
-	High	High	Low	Med	Med	Med	1.26	0.91	1.74	.1704	.3067	N	N
-	High	Low	High	Med	Med	Med	2.19	1.32	3.62	.0023	.0104	Y	Y
-	Low	High	High	Med	Med	Med	0.81	0.54	1.22	.3173	.4393	N	N
-	High	Low	Low	Med	Med	Med	0.94	0.59	1.48	.7834	.8058	N	N
-	Low	High	Low	Med	Med	Med	1.13	0.71	1.81	.5963	.6877	N	N
-	Low	Low	High	Med	Med	Med	0.81	0.59	1.12	.2060	.3339	N	N
-	Low	Low	Low	Med	Med	Med	0.69	0.49	0.96	.0279	.0837	Y	N

Note. Corr. Hyp=corresponding hypothesis.

Table S5

Models predicting varying thresholds of DSM-5 alcohol use disorder diagnosis from EXT, MDD, and ALC-sp PGS

	Mild or Higher (2+ Criteria)				Moderate or Higher (4+ Criteria)				Severe (6+ Criteria)			
Predictor	OR	95% CI	Z	*p*	OR	95% CI	Z	*p*	OR	95% CI	Z	*p*
	Model 1 (QIC=8415.32; QICu=8409.52)				Model 1 (QIC=8457.48; QICu=8457.00)				Model 1 (QIC=7422.91; QICu=7422.82)			
Intercept	1.68	0.92, 3.03	1.70	.09	**1.69**	**1.22, 3.06**	**-4.33**	**<.0001**	**0.13**	**0.08, 0.22**	**-7.61**	**<.0001**
Age	**0.98**	**0.97, 0.98**	**-13.16**	**<.0001**	**0.98**	**0.97, 0.98**	**-3.88**	**.0001**	1.00	0.99, 1.01	1.55	.12
Sex	**2.96**	**2.66, 3.32**	**19.68**	**<.0001**	**2.97**	**2.66, 3.32**	**19.24**	**<.0001**	**2.95**	**2.61, 3.33**	**17.58**	**<.0001**
EXT PGS	**1.45**	**1.36, 1.55**	**11.24**	**<.0001**	**1.53**	**1.43, 1.63**	**13.40**	**<.0001**	**1.51**	**1.41, 1.61**	**12.00**	**<.0001**
MDD PGS	1.05	0.99, 1.13	1.64	.10	**1.06**	**1.03, 1.09**	**2.53**	**.01**	**1.14**	**1.06, 1.21**	**3.81**	**0.0001**
ALC-sp PGS	**1.16**	**1.09, 1.36**	**5.14**	**<.0001**	**1.17**	**1.09, 1.22**	**7.65**	**<.0001**	**1.29**	**1.20, 1.37**	**7.51**	**<.0001**
	Model 2 (QIC=8419.03; QICu=8413.02)				Model 2 (QIC=8462.85; QICu=8461.69)				Model 2 (QIC=7428.06; QICu=7427.39)			
Intercept	1.70	0.94, 3.06	1.75	.08	**0.35**	**0.22, 0.57**	**-4.32**	**<.0001**	**0.13**	**0.08, 0.22**	**-7.61**	**<.0001**
Age	0.98	0.97, 0.98	**-13.14**	**<.0001**	**0.99**	**0.99, 1.00**	**-3.86**	**.0001**	1.00	0.99, 1.01	1.57	.12
Sex	2.96	2.66, 3.32	**19.68**	**<.0001**	**2.90**	**2.60, 3.23**	**19.25**	**<.0001**	**2.95**	**2.61, 3.33**	**17.57**	**<.0001**
EXT PGS	1.45	1.36, 1.55	**11.20**	**<.0001**	**1.52**	**1.43, 1.62**	**13.37**	**<.0001**	**1.51**	**1.41, 1.61**	**12.15**	**<.0001**
MDD PGS	1.05	0.99, 1.12	1.52	.13	**1.08**	**1.02, 1.15**	**2.58**	**.01**	**1.14**	**1.07, 1.22**	**3.83**	**0.0001**
ALC-sp PGS	1.16	1.09, 1.23	**5.17**	**<.0001**	**1.26**	**1.19, 1.34**	**7.66**	**<.0001**	**1.29**	**1.21, 1.38**	**7.48**	**<.0001**
EXT PGS*MDD PGS	0.98	0.93, 1.03	-0.74	.46	0.99	0.94, 1.05	-0.21	.83	1.01	0.95, 1.06	0.25	.80
EXT PGS*ALC-sp PGS	1.01	0.95, 1.07	0.39	.69	1.00	0.95, 1.06	0.10	.92	0.98	0.91, 1.04	-0.71	.48
MDD PGS*ALC-sp PGS	0.96	0.91, 1.02	-1.39	.16	0.97	0.91, 1.03	-1.05	.29	0.98	0.92, 1.05	-0.58	.56
	Model 3 (QIC=8420.93; QICu=8414.98)				Model 3 (QIC=8463.93; QICu=8462.67)				Model 3 (QIC=7428.22; QICu=7427.57)			
Intercept	1.70	0.94, 3.06	1.75	.08	**0.35**	**0.22, 0.57**	**-4.33**	**<.0001**	**0.13**	**0.08, 0.22**	**-7.62**	**<.0001**
Age	0.98	0.97, 0.98	**-13.14**	**<.0001**	**0.99**	**0.99, 1.00**	**-3.87**	**.0001**	1.00	0.99, 1.01	1.57	.12
Sex	2.96	2.66, 3.32	**19.66**	**<.0001**	**2.89**	**2.60, 3.23**	**19.24**	**<.0001**	**2.95**	**2.61, 3.33**	**17.56**	**<.0001**
EXT PGS	1.45	1.36, 1.55	**11.18**	**<.0001**	**1.52**	**1.43, 1.62**	**13.37**	**<.0001**	**1.51**	**1.41, 1.61**	**12.20**	**<.0001**
MDD PGS	1.05	0.99, 1.12	1.53	.13	**1.09**	**1.02, 1.16**	**2.67**	**.008**	**1.14**	**1.07, 1.22**	**3.96**	**<.0001**
ALC-sp PGS	1.16	1.09, 1.23	**5.02**	**<.0001**	**1.25**	**1.18, 1.33**	**7.27**	**<.0001**	**1.28**	**1.19, 1.37**	**7.07**	**<.0001**
EXT PGS*MDD PGS	0.98	0.93, 1.03	-0.73	.47	0.99	0.94, 1.05	-0.28	.78	1.00	0.95, 1.06	0.03	.97
EXT PGS*ALC-sp PGS	1.01	0.95, 1.07	0.40	.69	1.00	0.95, 1.06	0.10	.92	0.97	0.91, 1.04	-0.79	.43
MDD PGS*ALC-sp PGS	0.96	0.91, 1.02	-1.35	.18	0.97	0.91, 1.03	-1.02	.31	0.98	0.92, 1.04	-0.63	.53
EXT PGS*MDD PGS*ALC-sp PGS	1.01	0.96, 1.05	0.20	.84	1.03	0.97, 1.08	0.99	.32	1.04	0.98, 1.10	1.32	.19

Note. Ancestry PCs were included as covariates in all models but are not reported here for ease of presentation; female used as reference category for sex; bold indicates statistical significance, p<.05.

Table S6

Pairwise comparisons among individuals with any severity DSM-5 alcohol use disorder (2+ criteria) across levels of EXT PGS, MDD, and ALC-sp PGS

Outcomes	EXT PGS		Difference Test	MDD PGS		Difference Test	ALC-sp PGS		Difference Test
	High (N=1,403)	Low (N=1,050)	High EXT v. Low EXT	High (N=1,304)	Low (N=1,147)	High MDD v. Low MDD	High (N=1,319)	Low (N=1,136)	High v. Low
Psychopathology (Lifetime Diagnosis)	%	%	OR (95% CI)	%	%	OR (95% CI)	%	%	OR (95% CI)
DSM-IV CD	20.12%	10.53%	2.34 (1.79, 3.05)***	18.34%	11.21%	1.88 (1.44, 2.46)***	14.47%	15.98%	0.94 (0.72, 1.25)
DSM-IV ASPD	19.05%	10.61%	2.20 (1.69-2.87)***	16.73%	10.95%	1.89 (1.44, 2.48)***	14.91%	14.01%	1.14 (0.87, 1.48)
DSM-IV MDD	27.63%	23.48%	1.20 (0.96, 1.51)	31.73%	17.54%	2.23 (1.76, 2.82)***	25.52%	25.64%	1.01 (0.81, 1.27)
Criterion Count (Lifetime Maximum)	M (SD)	M (SD)	IRR (95% CI)	M (SD)	M (SD)	IRR (95% CI)	M (SD)	M (SD)	IRR (95% CI)
DSM-5 AUD	6.34 (3.23)	5.32 (3.08)	1.21 (1.15, 1.26)***	6.22 (3.29)	5.47 (3.09)	1.15 (1.09, 1.20)***	6.36 (3.27)	5.47 (3.09)	1.15 (1.10. 1.20)***
DSM-IV CD	1.39 (1.61)	0.97 (1.31)	1.46 (1.30, 1.63)***	1.32 (1.54)	0.97 (1.27)	1.38 (1.23, 1.53)***	1.17 (1.41)	1.15 (1.42)	1.04 (0.93, 1.26)
DSM-IV ASPD	2.98 (1.81)	2.20 (1.63)	1.39 (1.31, 1.47)***	2.84 (1.76)	2.40 (1.70)	1.21 (1.15, 1.29)***	2.71 (1.77)	2.63 (1.78)	1.05 (1.00, 1.11)
DSM-IV MDD	4.87 (3.75)	3.68 (3.57)	1.41 (1.17, 1.70)**	5.30 (3.56)	3.81 (3.63)	1.50 (1.27, 1.77)***	4.60 (3.74)	4.32 (3.76)	0.97 (0.81, 1.16)
Alcohol Expectancies	M (SD)	M (SD)	B (95% CI)	M (SD)	M (SD)	B (95% CI)	M (SD)	M (SD)	B (95% CI)
Phys/Soc Pleasure	32.59 (7.15)	32.48 (6.65)	0.08 (-0.55, 0.70)	32.20 (7.15)	33.02 (6.62)	-0.52 (-1.17, 0.13)	32.57 (6.88)	32.24 (6.90)	0.50 (-0.14, 1.14)
Arousal/Aggression	29.56 (7.28)	28.37 (6.89)	1.13 (0.46, 1.81)**	28.93 (7.32)	28.57 (6.88)	0.50 (-0.17, 1.16)	29.18 (7.04)	28.17 (7.13)	1.16 (0.52, 1.81)**
Social Assertiveness	35.24 (9.48)	34.09 (9.39)	1.06 (0.13, 1.99)*	34.67 (9.62)	34.45 (9.12)	0.41 (-0.52, 1.35)	34.93 (9.25)	33.89 (9.48)	1.21 (0.37, 2.06)*
Relaxation	30.41 (7.61)	29.38 (7.56)	0.86 (0.11, 1.61)*	29.95 (7.71)	29.78 (7.17)	0.39 (-0.32, 1.10)	30.20 (7.55)	29.37 (7.56)	0.98 (0.28, 1.68)*
Personality	M (SD)	M (SD)	B (95% CI)	M (SD)	M (SD)	B (95% CI)	M (SD)	M (SD)	B (95% CI)
Sensation Seeking	17.12 (4.37)	15.52 (5.31)	1.00 (-0.48, 2.48)	16.29 (4.50)	16.30 (5.08)	0.17 (-1.26, 1.60)	16.20 (4.92)	16.11 (4.66)	0.52 (-0,95, 1.98)
Neuroticism	55.39 (11.13)	53.40 (10.93)	1.84 (0.78, 2.90)**	56.06 (11.48)	52.64 (11.07)	3.30 (2.16, 4.43)***	54.72 (11.30)	54.34 (11.23)	0.57 (-0.58, 1.71)

Note. AUD: alcohol use disorder; CD: conduct disorder; ASPD: antisocial personality disorder; MDD: major depressive disorder; OR: odds ratio; IRR: incidence rate ratio; “low” used as reference category; difference tests derived from models that include age, sex, and ancestry principal components as covariates; ***p<.0001, **p<.001, *p<.05.

Table S7

Models predicting externalizing outcomes from combinations of EXT and MDD PGS among individuals with any severity DSM-5 alcohol use disorder (2+ criteria)

	CD Diagnosis				CD Criterion Count				AEQ Physical/Social Pleasure			
Predictor	OR	95% CI	Z	*p*	IRR	95% CI	Z	*p*	IRR	95% CI	Z	*p*
	Model 1 (QIC=2797.15; QICu=2800.66)				Model 1 (QIC=6002.10; QICu=6006.26)				Model 1 (QIC=3128.94; QICu=3131.00)			
Intercept	**0.50**	**0.27, 0.92**	**-2.22**	**.03**	**1.97**	**1.60, 2.42**	**6.40**	**<.0001**	**nr**	**nr**	**52.38**	**<.0001**
Age	**0.97**	**0.96, 0.97**	**-7.63**	**<.0001**	**0.98**	**0.98, 0.98**	**-11.16**	**<.0001**	**0.93**	**0.91, 0.95**	**-6.83**	**<.0001**
Sex	**3.07**	**2.47, 3.80**	**10.18**	**<.0001**	**1.77**	**1.63, 1.91**	**13.87**	**<.0001**	**2.60**	**1.60, 4.23**	**3.84**	**0.0001**
EXT PGS	**1.38**	**1.24, 1.54**	**5.87**	**<.0001**	**1.17**	**1.11, 1.22**	**6.65**	**<.0001**	1.09	0.83, 1.43	0.63	.53
MDD PGS	**1.19**	**1.07, 1.31**	**3.27**	**.001**	**1.10**	**1.05, 1.14**	**4.52**	**<.0001**	**0.70**	**0.54, 0.90**	**-2.75**	**0.006**
	Model 2 (QIC=2799.04; QICu=2802.59)				Model 2 (QIC=6003.62; QICu=6007.82)				Model 2 (QIC=3130.18; QICu=3132.00)			
Intercept	**0.50**	**0.27, 0.93**	**-2.19**	**.03**	**1.96**	**1.59, 2.42**	**6.33**	**<.0001**	**nr**	**nr**	**51.58**	**<.0001**
Age	**0.97**	**0.96, 0.97**	**-7.64**	**<.0001**	**0.98**	**0.98, 0.98**	**-11.15**	**<.0001**	**0.93**	**0.91, 0.95**	**-6.82**	**<.0001**
Sex	**3.06**	**2.47, 3.80**	**10.17**	**<.0001**	**1.77**	**1.63, 1.91**	**13.88**	**<.0001**	**2.59**	**1.59, 4.22**	**3.84**	**0.0001**
EXT PGS	**1.38**	**1.24, 1.54**	**5.90**	**<.0001**	**1.17**	**1.11, 1.22**	**6.58**	**<.0001**	1.09	0.83, 1.43	0.62	.53
MDD PGS	**1.19**	**1.07, 1.32**	**3.29**	**.001**	**1.10**	**1.05, 1.14**	**4.46**	**<.0001**	**0.70**	**0.54, 0.90**	**-2.75**	**0.006**
EXT PGS*MDD PGS	0.99	0.90, 1.09	-0.25	.80	1.00	0.97, 1.04	0.23	.82	1.05	0.83, 1.33	0.42	.67
	AEQ Arousal/Aggression				Sensation Seeking							
	IRR	95% CI	Z	*p*	IRR	95% CI	Z	*p*				
	Model 1 (QIC=3127.80; QICu=3129.00)				Model 1 (QIC=274.53; QICu=278.00)							
Intercept	**nr**	**nr**	**35.13**	**<.0001**	**nr**	**nr**	**5.55**	**<.0001**				
Age	0.98	0.96, 1.00	-1.61	.11	1.01	0.85, 1.20	0.14	.89				
Sex	1.12	0.67, 1.86	0.43	.67	**13.47**	**4.04, 45.05**	**4.24**	**<.0001**				
EXT PGS	**1.60**	**1.21, 2.13**	**3.26**	**0.001**	**1.76**	**1.00, 3.11**	**1.95**	**.05**				
MDD PGS	1.06	0.82, 1.38	0.46	.64	0.84	0.48, 1.49	-0.58	.56				
	Model 2 (QIC=3129.08; QICu=3130.00)				Model 2 (QIC=274.76; QICu=279.00)							
Intercept	**nr**	**nr**	**34.46**	**<.0001**	**nr**	**nr**	**5.66**	**<.0001**				
Age	0.98	0.96, 1.00	-1.59	.11	1.01	0.85, 1.20	0.13	.90				
Sex	1.11	0.67, 1.85	0.40	.69	**13.40**	**4.02, 44.69**	**4.22**	**<.0001**				
EXT PGS	**1.60**	**1.21, 2.12**	**3.26**	**.001**	1.73	0.98, 3.07	1.87	.06				
MDD PGS	1.06	0.82, 1.38	0.46	.64	0.85	0.48, 1.49	-0.57	.57				
EXT PGS*MDD PGS	1.23	0.96, 1.57	1.65	.10	0.91	0.58, 1.43	-0.4	.69				

Note. Ancestry PCs were included as covariates in all models but are not reported here for ease of presentation; female used as reference category for sex; nr: not reported due to excessively small size of exponentiated effect; bold indicates statistical significance, *p*<.05.

Table S8

Models predicting externalizing outcomes from combinations of EXT and MDD PGS among individuals with moderate to severe DSM-5 alcohol use disorder (4+ criteria)

	CD Diagnosis				CD Criterion Count				AEQ Physical/Social Pleasure			
Predictor	OR	95% CI	Z	*p*	IRR	95% CI	Z	*p*	IRR	95% CI	Z	*p*
	Model 1 (QIC=3677.84; QICu=3685.29)				Model 1 (QIC=2135.72; QICu=2140.44)				Model 1 (QIC=2049.36; QICu=2056.00)			
Intercept	**2.74**	**2.25, 3.33**	**9.98**	**<.0001**	0.80	0.41, 1.55	-0.66	.51	**nr**	**nr**	**45.47**	**<.0001**
Age	**0.98**	**0.97, 0.98**	**-11.36**	**<.0001**	**0.96**	**0.95, 0.97**	**-7.51**	**<.0001**	**0.93**	**0.91, 0.96**	**-5.22**	**<.0001**
Sex	**1.67**	**1.52, 1.83**	**11.07**	**<.0001**	**2.75**	**2.17, 3.49**	**8.30**	**<.0001**	1.88	0.97, 3.62	1.87	.06
EXT PGS	**1.12**	**1.06, 1.18**	**4.28**	**<.0001**	**1.26**	**1.12, 1.42**	**3.80**	**.0001**	1.03	0.72, 1.45	0.14	.89
MDD PGS	**1.09**	**1.04, 1.14**	**3.66**	**.0003**	**1.19**	**1.05, 1.33**	**2.82**	**.005**	0.77	0.55, 1.08	-1.49	.14
	Model 2 (QIC=3680.14; QICu=3687.62)				Model 2 (QIC=2137.62; QICu=2142.44)				Model 2 (QIC=2050.62; QICu=2057.00)			
Intercept	**2.73**	**2.24, 3.33**	**9.91**	**<.0001**	0.80	0.41, 1.55	-0.65	.51	**nr**	**nr**	**44.91**	**<.0001**
Age	**0.98**	**0.97, 0.98**	**-11.35**	**<.0001**	**0.96**	**0.95, 0.97**	**-7.51**	**<.0001**	**0.93**	**0.91, 0.96**	**-5.22**	**<.0001**
Sex	**1.67**	**1.52, 1.83**	**11.05**	**<.0001**	**2.75**	**2.17, 3.49**	**8.3**	**<.0001**	1.88	0.97, 3.64	1.88	.06
EXT PGS	**1.12**	**1.06, 1.18**	**4.18**	**<.0001**	**1.26**	**1.12, 1.42**	**3.76**	**.0002**	1.03	0.73, 1.46	0.17	.86
MDD PGS	**1.09**	**1.04, 1.14**	**3.50**	**.0005**	**1.19**	**1.05, 1.34**	**2.75**	**.006**	0.78	0.55, 1.10	-1.42	.16
EXT PGS*MDD PGS	1.01	0.96, 1.05	0.34	.74	1.00	0.90, 1.11	-0.03	.98	0.92	0.67, 1.27	-0.5	.61
	AEQ Arousal/Aggression				Sensation Seeking							
	IRR	95% CI	Z	*p*	IRR	95% CI	Z	*p*				
	Model 1 (QIC=2048.55; QICu=2054.00)				Model 1 (QIC=136.31; QICu=145.00)							
Intercept	**nr**	**nr**	**33.86**	**<.0001**	**nr**	**nr**	**5.53**	**<.0001**				
Age	0.98	0.96, 1.01	-1.17	.24	0.89	0.71, 1.13	-0.94	.35				
Sex	0.71	0.38, 1.35	-1.04	.30	**5.00**	**0.98, 25.54**	**1.93**	**.05**				
EXT PGS	1.37	0.97, 1.93	1.78	.08	1.52	0.65, 3.54	0.97	.33				
MDD PGS	0.90	0.64, 1.24	-0.66	.51	0.97	0.50, 1.90	-0.09	.93				
	Model 2 (QIC=2049.51; QICu=2055.00)				Model 2 (QIC=136.61; QICu=146.00)							
Intercept	**nr**	**nr**	**33.67**	**<.0001**	**nr**	**nr**	**5.43**	**<.0001**				
Age	0.98	0.96, 1.01	-1.15	.25	0.90	0.71, 1.14	-0.88	.38				
Sex	0.71	0.38, 1.34	-1.06	.29	5.06	**0.97, 26.42**	**1.92**	**.05**				
EXT PGS	1.35	0.96, 1.91	1.73	.08	1.57	0.69, 3.55	1.08	.28				
MDD PGS	0.88	0.63, 1.22	-0.76	.44	0.95	0.46, 1.95	-0.15	.88				
EXT PGS*MDD PGS	1.16	0.86, 1.57	0.97	.33	1.08	0.59, 2.01	0.26	.80				

Note. Ancestry PCs were included as covariates in all models but are not reported here for ease of presentation; female used as reference category for sex; nr: not reported due to excessively small size of exponentiated effect; bold indicates statistical significance, *p*<.05.

Table S9

Models predicting internalizing outcomes from combinations of EXT and MDD PGS among individuals with any severity DSM-5 alcohol use disorder (2+ criteria)

	MDD Diagnosis				MDD Criterion Count				AEQ Social Assertiveness			
Predictor	OR	95% CI	Z	*p*	IRR	95% CI	Z	*p*	IRR	95% CI	Z	*p*
	Model 1 (QIC=3364.45; QICu=3366.50)				Model 1 (QIC=-8726.32; QICu=-8722.94)				Model 1 (QIC=3129.01; QICu=3129.00)			
Intercept	0.63	0.34, 1.16	-1.48	.14	**2.83**	**1.79, 4.46**	**4.47**	**<.0001**	**nr**	**nr**	**31.82**	**<.0001**
Age	**0.99**	**0.98, 1.00**	**-3.30**	**.001**	**1.02**	**1.01, 1.04**	**3.05**	**.002**	**0.94**	**0.92, 0.97**	**-4.15**	**<.0001**
Sex	**0.35**	**0.29, 0.42**	**-11.69**	**<.0001**	**0.61**	**0.54, 0.70**	**-7.53**	**<.0001**	1.35	0.69, 2.63	0.88	.38
EXT PGS	1.05	0.96, 1.16	1.06	.29	1.06	0.98, 1.14	1.53	.13	**1.48**	**1.01, 2.17**	**2.01**	**.04**
MDD PGS	**1.38**	**1.25, 1.52**	**6.60**	**<.0001**	**1.17**	**1.08, 1.26**	**4.07**	**<.0001**	0.98	0.69, 1.39	-0.12	.91
	Model 2 (QIC=3366.09; QICu=3368.38)				Model 2 (QIC=-8717.01; QICu=-8713.59)				Model 2 (QIC=3130.01; QICu=3130.00)			
Intercept	0.62	0.33, 1.16	-1.50	.13	**2.81**	**1.77, 4.45**	**4.41**	**<.0001**	**nr**	**nr**	**31.2**	**<.0001**
Age	**0.99**	**0.98, 1.00**	**-3.29**	**.001**	**1.02**	**1.01, 1.04**	**3.05**	**.002**	**0.94**	**0.92, 0.97**	**-4.13**	**<.0001**
Sex	**0.35**	**0.29, 0.42**	**-11.69**	**<.0001**	**0.61**	**0.54, 0.70**	**-7.54**	**<.0001**	1.34	0.69, 2.60	0.86	.39
EXT PGS	1.05	0.96, 1.15	1.02	.31	1.06	0.99, 1.14	1.56	.12	**1.48**	**1.01, 2.16**	**2.02**	**.04**
MDD PGS	**1.38**	**1.25, 1.51**	**6.59**	**<.0001**	**1.17**	**1.08, 1.26**	**4.03**	**<.0001**	0.98	0.69, 1.39	-0.12	.91
EXT PGS*MDD PGS	1.01	0.94, 1.10	0.36	.72	1.01	0.95, 1.08	0.36	.72	**1.35**	**1.00, 1.82**	**1.96**	**.05**
	AEQ Relaxation/Tension Reduction				Neuroticism							
	IRR	95% CI	Z	*p*	IRR	95% CI	Z	*p*				
	Model 1 (QIC=3124.01; QICu=3125.00)				Model 1 (QIC=3120.71; QICu=3122.00)							
Intercept	**nr**	**nr**	**35.22**	**<.0001**	**nr**	**nr**	**36.26**	**<.0001**				
Age	**0.97**	**0.94, 0.99**	**-2.94**	**.003**	**0.91**	**0.88, 0.94**	**-5.81**	**<.0001**				
Sex	**4.29**	**2.47, 7.45**	**5.16**	**<.0001**	0.70	0.31, 1.58	-0.86	.39				
EXT PGS	**1.37**	**1.00, 1.87**	**1.94**	**.05**	**1.57**	**1.03, 2.38**	**2.10**	**.04**				
MDD PGS	1.05	0.80, 1.38	0.35	.72	**4.28**	**2.74, 6.67**	**6.40**	**<.0001**				
	Model 2 (QIC=3125.04; QICu=3126.00)				Model 2 (QIC=3121.82; QICu=3123.00)							
Intercept	**nr**	**nr**	**34.69**	**<.0001**	**nr**	**nr**	**35.85**	**<.0001**				
Age	**0.97**	**0.94, 0.99**	**-2.91**	**.004**	**0.91**	**0.88, 0.94**	**-5.80**	**<.0001**				
Sex	**4.26**	**2.45, 7.40**	**5.15**	**<.0001**	0.70	0.31, 1.56	-0.88	.38				
EXT PGS	**1.36**	**1.00, 1.87**	**1.94**	**.05**	**1.56**	**1.03, 2.37**	**2.09**	**.04**				
MDD PGS	1.05	0.80, 1.38	0.35	.72	**4.27**	**2.74, 6.65**	**6.41**	**<.0001**				
EXT PGS*MDD PGS	1.20	0.94, 1.54	1.44	.15	1.40	0.96, 2.04	1.77	.08				

Note. Ancestry PCs were included as covariates in all models but are not reported here for ease of presentation; female used as reference category for sex; bold indicates statistical significance, *p*<.05.

Table S10

Models predicting internalizing outcomes from combinations of EXT and MDD PGS among individuals with DSM-5 moderate to severe alcohol use disorder (4+ criteria)

	MDD Diagnosis				MDD Criterion Count				AEQ Social Assertiveness			
Predictor	OR	95% CI	Z	*p*	IRR	95% CI	Z	*p*	IRR	95% CI	Z	*p*
	Model 1 (QIC=-4987.45; QICu=-4983.93)				Model 1 (QIC=2170.83; QICu=-2174.86)				Model 1 (QIC=2048.53; QICu=2054.00)			
Intercept	**4.24**	**2.31, 7.78**	**4.67**	**<.0001**	0.55	0.26, 1.19	-1.51	.13	**nr**	**nr**	**33.87**	**<.0001**
Age	1.01	0.99, 1.04	1.3	.19	0.99	0.98, 1.00	-1.78	.07	**0.96**	**0.93, 1.00**	**-2.19**	**.03**
Sex	**0.62**	**0.53, 0.73**	**-5.93**	**<.0001**	**0.32**	**0.26, 0.40**	**-10.28**	**<.0001**	0.91	0.40, 2.05	-0.23	.81
EXT PGS	1.00	0.91, 1.10	0.06	.95	1.05	0.93, 1.18	0.81	.42	1.41	0.90, 2.23	1.49	.14
MDD PGS	**1.15**	**1.05, 1.24**	**3.23**	**.001**	**1.31**	**1.17, 1.47**	**4.57**	**<.0001**	0.90	0.59, 1.38	-0.49	.63
	Model 2 (QIC=-4967.18; QICu=-4963.39)				Model 2 (QIC=2171.61; QICu=2176.01)				Model 2 (QIC=2049.56; QICu=2055.00)			
Intercept	**4.28**	**2.32, 7.88**	**4.67**	**<.0001**	0.57	0.26, 1.21	-1.46	.14	**nr**	**nr**	**33.61**	**<.0001**
Age	1.01	0.99, 1.04	1.3	0.19	0.99	0.98, 1.00	-1.82	.07	**0.96**	**0.93, 1.00**	**-2.16**	**.03**
Sex	**0.62**	**0.53, 0.73**	**-5.89**	**<.0001**	**0.33**	**0.26, 0.40**	**-10.24**	**<.0001**	0.90	0.40, 2.02	-0.26	.79
EXT PGS	1.00	0.91, 1.10	0.02	0.99	1.06	0.94, 1.19	0.96	.34	1.39	0.89, 2.18	1.44	.15
MDD PGS	**1.15**	**1.06, 1.25**	**3.23**	**0.001**	**1.32**	**1.18, 1.49**	**4.66**	**<.0001**	0.87	0.57, 1.33	-0.63	.53
EXT PGS*MDD PGS	0.99	0.92, 1.06	-0.32	0.75	0.95	0.86, 1.05	-1.02	.31	1.28	0.86, 1.90	1.21	.23
	AEQ Relaxation/Tension Reduction				Neuroticism							
	IRR	95% CI	Z	*p*	IRR	95% CI	Z	*p*				
	Model 1 (QIC=2044.84; QICu=2050.00)				Model 1 (QIC=2031.18; QICu=2035.00)							
Intercept	**nr**	**nr**	**32.35**	**<.0001**	**nr**	**nr**	**33.47**	**<.0001**				
Age	**0.96**	**0.94, 0.99**	**-2.43**	**.012**	**0.92**	**0.88, 0.96**	**-4.00**	**<.0001**				
Sex	**2.88**	**1.41, 5.88**	**2.9**	**.004**	0.51	0.18, 1.44	-1.28	.20				
EXT PGS	1.32	0.91, 1.92	1.45	.15	1.10	0.65, 1.86	0.34	.73				
MDD PGS	1.08	0.76, 1.54	0.45	.65	**2.95**	**1.71, 5.07**	**3.90**	**<.0001**				
	Model 2 (QIC=2045.97; QICu=2051.00)				Model 2 (QIC=2032.24; QICu=2036.00)							
Intercept	**nr**	**nr**	**32.2**	**<.0001**	**nr**	**nr**	**33.23**	**<.0001**				
Age	**0.96**	**0.94, 0.99**	**-2.42**	**.02**	**0.92**	**0.88, 0.96**	**-3.97**	**<.0001**				
Sex	**2.87**	**1.40, 5.88**	**2.89**	**.004**	0.50	0.18, 1.41	-1.31	.19				
EXT PGS	1.32	0.91, 1.91	1.44	.15	1.06	0.63, 1.79	0.21	.83				
MDD PGS	1.08	0.76, 1.54	0.42	.67	**2.76**	**1.60, 4.78**	**3.64**	**.0003**				
EXT PGS*MDD PGS	1.02	0.73, 1.43	0.14	.89	**1.63**	**1.00, 2.65**	**1.98**	**.05**				

Note. Ancestry PCs were included as covariates in all models but are not reported here for ease of presentation; female used as reference category for sex; bold indicates statistical significance, *p*<.05.

Figure S1

Odds ratios (ORs) of pairwise comparisons of DSM-5 alcohol use disorder diagnosis (2+ criteria threshold) across 9 PGS classification groups

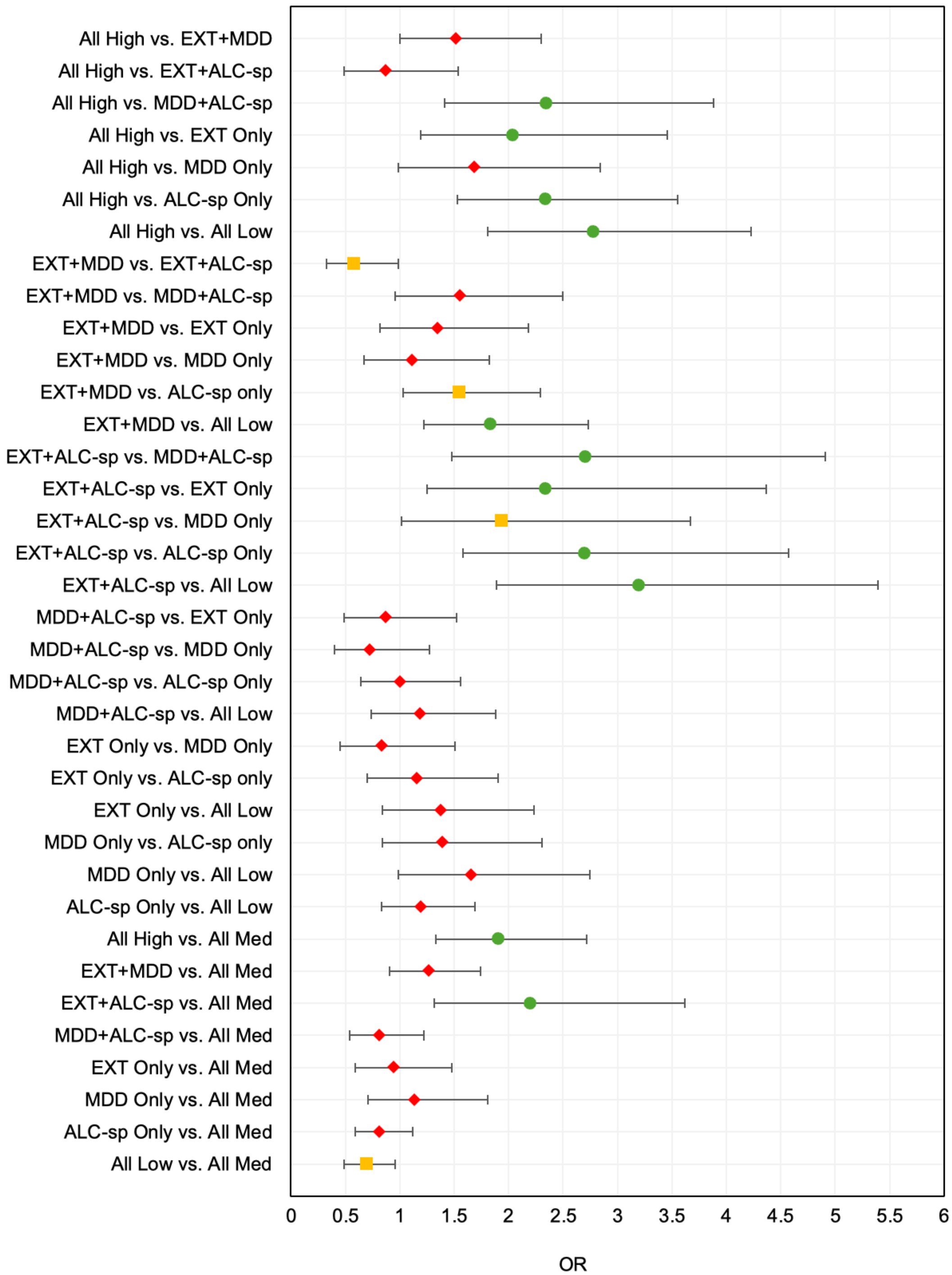

Note. Green circles denote statistical significance; yellow squares denote statistical significance before, but not after, FDR correction; red diamonds denote nonsignificance.
